# Vital evidence: Change in the marine ^14^C reservoir around New Zealand (Aotearoa) and implications for the timing of Polynesian settlement

**DOI:** 10.1038/s41598-020-70227-3

**Published:** 2020-08-31

**Authors:** Fiona Petchey, Magdalena M. E. Schmid

**Affiliations:** 1 Radiocarbon Dating Laboratory, Division of Health, Engineering, Computing and Science, University of Waikato, 3240 Hamilton, New Zealand; 2 ARC Centre of Excellence for Australian Biodiversity and Heritage, College of Arts, Society and Education, James Cook University, PO Box 6811, Cairns, QLD 4870 Australia; 3 Institute of Pre- and Protohistoric Archaeology, Christian-Albrechts-Universität zu Kiel, 24118 Kiel, Germany

**Keywords:** Palaeoceanography, Climate-change adaptation, Climate-change impacts

## Abstract

Precise and accurate radiocarbon chronologies are essential to achieve tight chronological control for the ~ 750-years since Polynesian settlement of New Zealand. This goal has, however, been elusive. While radiocarbon datasets in the region are typically dominated by marine and estuarine shell dates, such chronological information has been ignored by those interpreting the timing of key events because a detailed regional calibration methodology for marine shell, comparable to the highly precise Southern Hemisphere calibration curve, is lacking. In this paper, we present the first temporal ^14^C marine offset (Δ*R*) model for New Zealand based on paired estuarine/marine and terrestrial radiocarbon dates from 52 archaeological contexts. Our dataset displays significant offsets between the measured New Zealand data and the modelled global marine radiocarbon curve. These shifts are associated with oceanographic fluctuation at the onset of the Little Ice Age ~ AD 1350–1450 (650–500 BP). The application of a regional and temporal correction to archaeological shell dates provides complimentary information to terrestrial radiocarbon production and has the potential to add structure to the blurred chronology that has plagued archaeological theories about the colonization of New Zealand, and other Pacific islands, for decades.

## Introduction

The short prehistory of human occupation of New Zealand (NZ) has been vigorously debated for many decades. Recent research suggests that colonization started in the mid to late thirteenth century AD^1,2^, but that sustained, widespread settlement was later^3,4^. Walter et al.^5^ have argued that an apparent strong archaeological (i.e. radiocarbon [^14^C]) signal in the early fourteenth century is evidence of a mass migration event^5^, but this is not universally accepted^6^. By the mid-fifteenth century, moa (large flightless birds) had become extinct in the North Island with the last remnant populations soon dying out in the South Island^4,7^. During this time, settlement had expanded from sheltered coastal locations into inland regions and the extent and intensity of gardening increased^8,9^. By the end of the fifteenth century, fortified settlements (pā) began to be built across the landscape for reasons that are not yet clear^10^. These events most likely occurred at different rates in different places across NZ. Artefacts that document adaptation to new tasks, environments and materials often display regional variation, before eventually transforming into traditional Māori styles^11,12^. Despite thousands of published ^14^C dates, the middle, or transitional phase of Māori archaeology (~ AD 1450 to AD 1650), has been described by Anderson (p.7)^9^ as a “shadowland between highlights of Polynesian colonisation and classic Māori culture”. A lack of ^14^C precision over a “particularly wiggly portion of the radiocarbon calibration curve” is commonly cited as the limiting factor^5^.

Marine shell remains an important material for ^14^C dating in the Pacific because it is common in archaeological sites and is easy to identify. In fact, 62% of the NZ archaeological materials dated at the Radiocarbon Dating Laboratory, University of Waikato, are marine/estuarine shell (as of Nov 2019). This equates to ~ 1800 shell dates for the NZ sequence. Regional chronological studies^2–4^ have, however, concentrated on terrestrial materials that make up but a fraction of all available dates. In order to complement and enhance these terrestrial studies, we need greater clarity on what ^14^C marine reservoir correction value to use. The standard approach for the last ~ 30 years has used the modelled global surface marine curve (e.g., Marine13^14^) determined from a box-diffusion model of global carbon exchange derived largely from ^14^C atmospheric data. This curve corrects for the approximate 400-year difference between terrestrial and marine ^14^C reservoirs. A local ‘reservoir offset’ (Δ*R*) is applied to the marine curve to account for regional variation^15^. The Δ*R* for a specific location ‘(s)’ is calculated using the formula *R*s(t) – *R*g(t) = Δ*R*(s), where (Δ*R*(s)) is the difference between the global average (*R*g(t)) and the actual ^14^C activity of the surface ocean at a particular location (*R*s(t); i.e., the archaeological marine date) at that particular time (based on the terrestrial ^14^C date or another independent means by which to determine age, such as U-Th dates). This methodology assumes that the calibration curve accounts for temporal variation.

**Figure 1:**
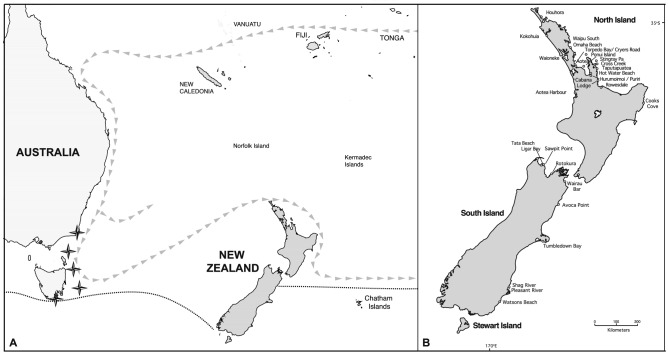
(**A**) Southwest Pacific Ocean showing location of black coral sampling locations (star) in relation to New Zealand. Grey arrows indicate direction of surface currents of the Subtropical Gyre. Dotted line indicates the approximate location of the Subtropical Front. (**B**) Location of archaeological sites mentioned in the text.

Some marine Δ*R* work has been carried out around the NZ coastline^13,16–19^ using ‘modern’ (pre-AD 1950) shellfish of known collection date. Petchey et al.^19^ identified regional variation between the different outlying islands—Norfolk, Kermadec and Chatham islands—because of the complex interplay of currents and water bodies (the Tasman Sea and the South Pacific Ocean). They settled on an average Δ*R*-value of − 7 ± 45 ^14^C years for NZ as a whole, with little discernible variation around the main coastline, but identified a much higher and variable Δ*R* for the Chatham Islands caused by upwelling of ^14^C-depleted water along the Chatham Rise and Subtropical Front (which skirts the southeast coast of the South Island) (Fig. 1a). The identification of temporal change has been more difficult to evaluate because of the low precision and limited numbers of archaeological ^14^C dates used, but the general consensus was that the regional ocean around NZ appears to have been stable over the recent past^17,18^.

This assumption of temporal stability in the marine reservoir offset is problematic. Recent ^14^C values of independently U-Th dated black coral ^14^C ages from Tasmania (Fig. 1a)^20^ indicate significant reservoir shifts. These shifts are also evident in archaeological Δ*R* from central South Pacific islands^21^ and have major implications for archaeological chronologies that rely on marine and estuarine shell^22^. It is likely that NZ waters are similarly affected, though this has not yet been quantified. In particular, the Tasmanian black coral data show a major positive Δ*R* shift, up to + 150 ^14^C years, between ca. 400–200 years BP (AD 1550–1750) and a negative shift of up to − 200 ^14^C years around 600 years BP (AD 1350). These Δ*R* offsets would introduce hundreds of years of offset into shell ^14^C dates from NZ archaeological contexts, contributing to the blurring of calibrated shell ages. A complex interplay of climate and oceanographic conditions is likely responsible for these shifts, which will almost certainly have had an impact on human adaptation to NZ and may, in part, have been responsible for key economic changes evident in the excavated material remains. Recent revisions to the global marine ^14^C curve^23,24^ enable regional calibration options, but these options are based on climate models and not on measured data and do not come close to the resolution of measured terrestrial curves such as SHCal13^25^. Contained within the archaeological literature are a host of ‘paired’ (i.e., from the same context) shell and charcoal ^14^C dates which can help evaluate the extent of this problem for NZ.

In this paper, we present a temporal Δ*R* model for NZ based on published archaeological paired estuarine/marine and terrestrial ^14^C dates spanning the entire sequence since initial Polynesian settlement. This dataset is ten times larger than any previous regional chronological assessment, and is in close agreement with black coral ^14^C data from Tasmania. Both archaeological and black coral datasets document significant changes in Δ*R* over the last 750 years which attest to changes in the ocean ^14^C reservoir associated with major climatic events.

## Methods

The marine reservoir age ‘*R*’ is the offset in ^14^C age between the atmosphere and the global ocean. Regional offsets from *R* are termed the ‘local marine reservoir age’, or ‘Δ*R*’^15^. Calibration of marine ^14^C dates involves application of a Δ*R*-value to the marine calibration curve (e.g., Marine13^14^) to account for these regional offsets. A Δ*R* can be calculated from terrestrial and marine samples excavated from archaeological sites. A regional reservoir offset can also be calculated from known-age marine carbonates collected prior to atmospheric bomb testing^19^, or from samples where independently measured calendar ages can be obtained, such as coral dated by both U-Th and ^14^C^20^. No matter what materials are used to determine Δ*R*, they must comply with a set of prerequisites^26^. For archaeological shell samples, the age is determined by dating short-lived (must be identified to species and/or element), ‘paired’ terrestrial materials from contemporaneous contexts.

A careful evaluation of each context and set of paired dates from each archaeological context was made prior to inclusion in our temporal model of changing marine Δ*R*. During this evaluation of legacy data, we found numerous issues that warrant mention, as indicated below.

### Calibration issues

Any terrestrial sample with a calibrated age of less than 200 BP produces multiple calibrated ages because rapid fluctuations in ^14^C have resulted in ‘wiggles’ in the calibration curve. In these regions it is near impossible to evaluate which of the multiple wiggles is the true age of the sample, and therefore what the exact marine offset is (Fig. S1). Consequently, any samples with a calibrated mean age of less than 200 BP is of limited use to our evaluation.

### Context uncertainty

Typically, the most robust determinations come from well-defined features. We found the selection of charcoal and shell dates from single contexts was rare, with the majority of dates sourced from broader ‘midden’ layers. Radiocarbon dates from horticultural soils have not been included because these are typically mixed (plaggen soils)^27^. Where more than one terrestrial date was available from the same context we used the Chi square test to insure the ^14^C results were indistinguishable (multiple terrestrial dates were only identified from thirteen contexts).

Differences between terrestrial and marine ^14^C dates have previously led researchers to search for clues as to the cause of these offsets. Anomalies have been attributed to hardwater, taxa variation, upwelling, inbuilt age in charcoal and the incorporation of natural shell or reuse of previous midden material^12,28,29^. Often the integrity of the archaeological feature has been questioned on the basis of divergent terrestrial and marine ^14^C results, even when there is limited evidence of disturbance. Certainly, disturbance remains a very strong candidate for apparent Δ*R* variation with both positive and negative Δ*R* values possible. In this study, we have ignored any pre-conceived assumptions of site disturbance, upwelling, taxa variation and/or hardwater impact, unless there is additional independent evidence to back up these claims.

### Material suitability

The literature is full of recommendations regarding the suitability of material for ^14^C dating and different pretreatment methodologies used. As the science progresses, many of these recommendations have been re-evaluated. With this in mind, we have carefully assessed preconceived ideas regarding the value of different sample types and different pretreatments.

All charcoal dates included in this evaluation have been identified to species that are considered suitable for ^14^C dating. Unfortunately, it is rare for the original publications to specifically state if the material has been positively identified as small diameter twigs, and there are very few dates on seeds. Anderson^29^ has speculated that even “short-lived” charcoal may have inbuilt age of 50–150 years, but this is difficult to evaluate for each pair of dates. Inbuilt age reduces the apparent age difference between the terrestrial and marine proxies and results in a more negative Δ*R*. Delamination of growth rings from larger branches during combustion is also possible, while differences have been noticed between the ages of twigs from shrubby plants and twigs from larger trees (W. Gumbley, *pers. comm*., Nov 2019). We have excluded all dates on bark because the rate of shredding and accumulation varies depending on taxa^30^. We have also excluded dates on tree fern (ponga) because they grow in a spiral pattern making it difficult to distinguish early from late growth (R. Wallace, *pers. comm*., Nov 2019).

Most shellfish precipitate their shells in equilibrium with the isotopic signature of dissolved inorganic carbon from the waters they live in^31^. Terrestrial organic material from rivers or rainwater runoff generally only have a small negative impact on the Δ*R* of filter-feeding estuarine bivalves because the uptake of this carbon is typically less than 10% of the 400-year difference between the marine and terrestrial ^14^C reservoirs^32^. Areas with calcareous rocks can, however, result in very positive Δ*R* values (termed a hardwater effect) because the shellfish uptake ancient bicarbonate ions that percolate through the substrate. Typically, this is highly localised and the presence of limestone does not guarantee a hardwater effect in suspension feeding bivalves^32^. Deposit-feeding and herbivorous shellfish can ingest both young and old (‘stored’) carbon which may have a significant impact on ^14^C ages in areas with limestone or old sediment^33^. In the NZ context, the deposit-feeder *Amphibola crenata* is the best studied example of this problem^34^, and dates of this taxa are excluded from our evaluation. All other shellfish taxa were evaluated on a individual basis. Although upwelling has been suggested as a cause for some anomalous shell ages there is no definitive evidence of any such influence on marine shell along the NZ coastline^19,35^.

Bone dating has a long and troubled history in the dating of archaeological contexts. Problems related to bone pretreatment and diet variability make this material incredibly complex to interpret. For this reason, the following constraints have been applied:

1. All ‘collagen’ dates are excluded (based on recommendations by Petchey^36^). This definition includes any bones that were only acid washed, or acid and base washed. Samples pretreated to gelatin or ultrafiltered gelatin are accepted^34,36^ except where the laboratory has identified an inhouse problem^37^.
2. Tripeptide dates on fish bone^38^ are excluded following research at the Oxford Radiocarbon laboratory that indicated column bleed could result in the introduction of older carbon (Tom Higham, *pers comm*., Oct 2019).
3. *Rattus exulans* ^14^C dates are excluded. In the mid 1990′s there was considerable debate over laboratory errors that may have resulted in anomalous *R. exulans* dates^39^. These errors are likely to reflect the limitations of accelerator mass spectrometry ^14^C dating processes at a time when these extremely tiny samples pushed the limits of the technique. Technical improvements and stringent quality control procedures now used by many laboratories limit a repeat of this episode.
4. We have included bird bone dates from terrestrial or marine environments, but birds that inhabit freshwater lakes and rivers are excluded due to dietary complexity^40^.

### Δ*R* calculation

Δ*R* values for both ^14^C and U-Th dated pairs (Table S1) have been calculated using the online tool found at https://calib.org/deltar/^41^. This calibrates the terrestrial ^14^C age with the appropriate calibration curve and then reverse-calibrates discrete points of the resulting probability density function with the marine calibration curve (Marine13^14^). We have used the Southern Hemisphere calibration curve (SHCal13^25^) for terrestrial samples. Calendar ages derived from U-Th measurements from Komugabe-Dixson et al.^20^ are similarly reverse-calibrated using the marine calibration curve.

### Construction of the ‘New Marine’ regional calibration curve

We have created a regional curve for use in OxCal^42^ using published U-Th and ^14^C dates from coral sequences reported by Komugabe-Dixson et al.^20^ An OxCal curve file (0.14c) was constructed from the calendar age, derived from the mean U-Th age, the ^14^C age in BP and the standard uncertainty on ^14^C age. This mock curve is based on limited regional inter-comparison and has not undergone rigorous statistical evaluation.

## Results

We have identified 52 Δ*R* marine/terrestrial pairs recovered from contemporaneous deposits within 36 archaeological sites (Table S1). The majority of sites are located on the northwest coast of the North Island; the Coromandel and Auckland regions of NZ (Fig. 1b). There is an absence of material from the west coast of the South Island. Twelve contexts have a single terrestrial and a single marine date, while twenty-five contexts have a single terrestrial date for comparison with multiple marine dates. This limits assessment of deposit integrity, here determined by statistical agreement between terrestrial ^14^C dates. Thirteen sites contain multiple terrestrial and multiple marine dates. These provide the most robust evaluation and the ability to spot anomalies caused by taphanomic, environmental, taxa specific and/or laboratory variables (Table S2).

**Figure 2:**
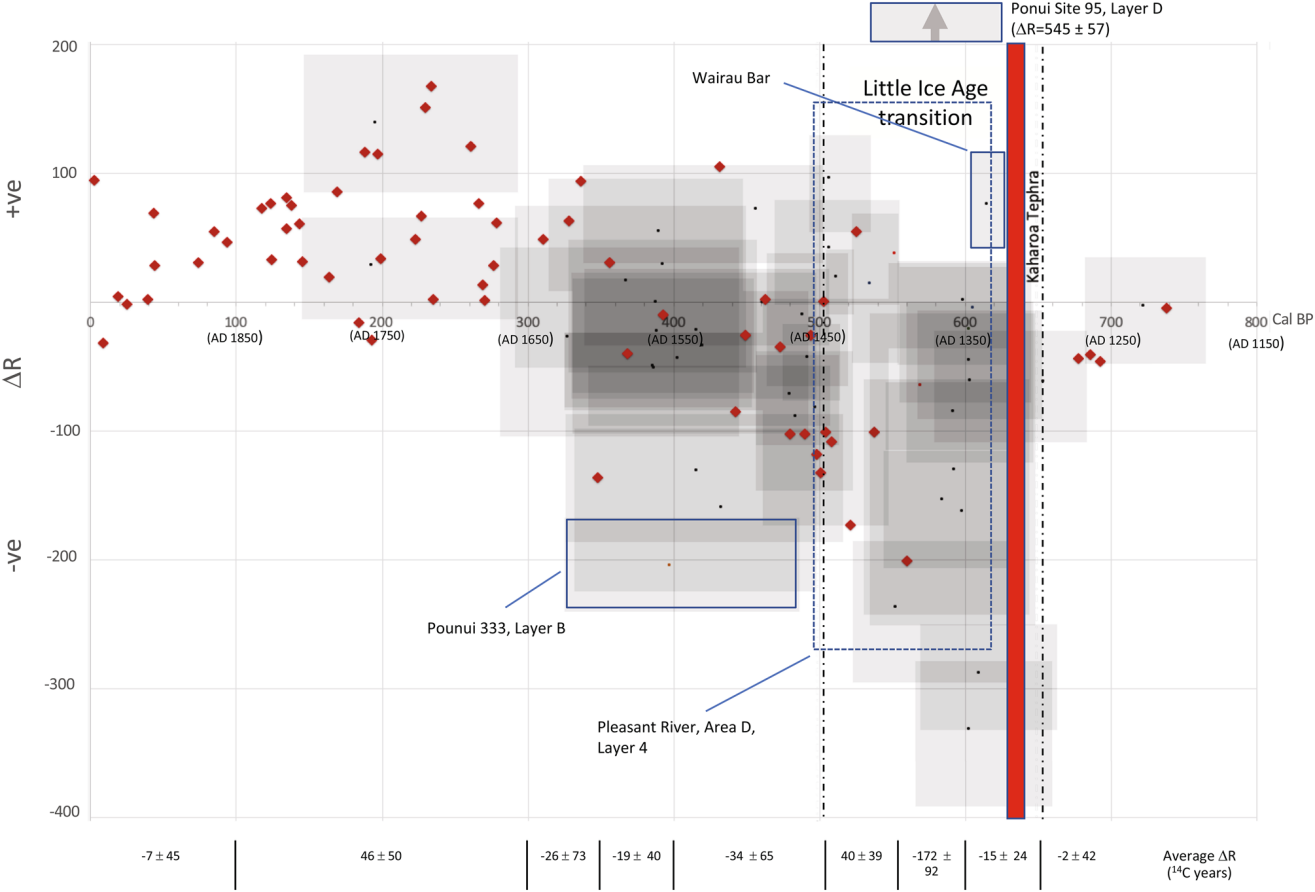
Temporal marine Δ*R* variation between 0 and 800 cal BP. Red diamonds = U-Th/^14^C ages for Tasmania^20^ (error bars not shown). Grey boxes = calibrated terrestrial age range for each site (horizontal) by Δ*R* error (vertical) (reported at 68.2% probability and calculated using https://calib.org/deltar/) ^41^. Black dots = median calibrated terrestrial age by mean Δ*R*-value for each archaeological site (values given in Table S1). Results from sites outlined by boxes are excluded from the temporal average Δ*R*-value reported in Table S2. Only archaeological sites with a median age > 200 cal BP shown. Dashed lines show period of ‘Little Ice Age’ transition^43^. Age of the Kaharoa Tephra (red bar) is based on Hogg et al.^44^ (see Text S1 for information on the sites highlighted).

Black coral and archaeological Δ*R* values are plotted in Fig. 2. Both sets display the same trend whereby the Δ*R* before 600 BP (AD 1350) is slightly negative, followed by an extreme negative shift around 600 BP and a subsequent gradual return to more positive values around 500 BP (AD 1450). This positive Δ*R* is very brief and quickly returns to moderately negative values which remain relatively stable over the next 150 years. Between 0 and 350 BP (AD 1600 and 1950) ΔR values are more positive. The U-Th dated black corals produce tightly controlled calendar ages, but there are relatively few values between 750 and 500 cal BP and none between 670 and 554 cal BP. The terrestrial dates for each archaeological context have a wider spread in calendar age but this additional information enhances the extant coral data and may more closely represent the NZ situation than coral from Tasmania.

We suspect that the black coral and archaeological datasets only hint at the complexity of the marine ^14^C environment. Additional research is required to map the temporal and geographic variability of the marine reservoir around NZ but, in the meantime, we have calculated the following Δ*R* offsets between 0 and 750 cal BP using a combination of the black coral dataset, modern pre-AD 1950 shellfish, and the archaeological pairs:

1. Between 0 and 100 cal BP we recommend the use of − 7 ± 45 ^14^C years derived from modern pre-AD 1950 shellfish (after Petchey et al. 2008).
2. Between 100 and 300 cal BP a value of 46 ± 50 ^14^C years reflects a combination of black coral and archaeological Δ*R* (χ^2^_27:0.05_ = 31.49 < 40.11).
3. For the rest of the NZ sequence (i.e., 750–300 cal BP), the following archaeological Δ*R* have been calculated for each temporal block: 700–650 cal BP = − 2 ± 42 ^14^C years; 650–600 cal BP = − 15 ± 24 ^14^C years; 600–550 cal BP = − 172 ± 92 ^14^C years; 550–500 cal BP = 40 ± 39 ^14^C years; 500–400 cal BP = − 34 ± 65 ^14^C years; 400–350 cal BP = − 19 ± 40 ^14^C years; and 350–300 cal BP = − 26 ± 73 ^14^C years (see Table S2 and Fig. 2).

**Figure 3:**
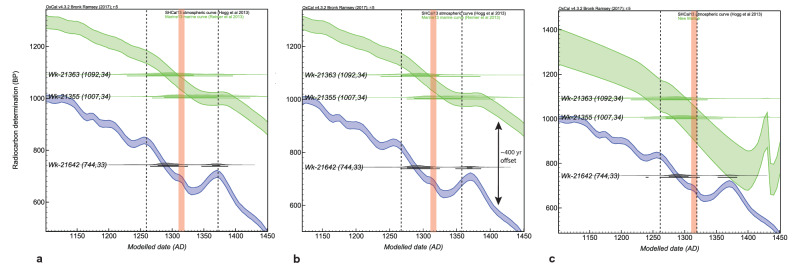
Calibrated radiocarbon dates from Cross Creek Layer 9 grouped into a single phase in OxCal^46^. (**A**) Using the: (**A**) pre-AD 1950 marine Δ*R* of − 7 ± 45 ^14^C yrs^19^; (**B**) Δ*R* of − 15 ± 24 ^14^C years based on archaeological data collected in this paper; and (**C**) Age calibrated using the black coral dataset (‘New Marine’ calibration curve). The extended sequence analysis of layers 7 and 9 is presented in Fig. S2. Vertical dashed lines are the 68% prob. maximum age ranges of the marine dates (Wk-21363 and Wk-21355). Wk-21642 is a terrestrial date. The red line represents the calendar age of the Kaharoa tephra^44^. Green band = marine calibration curve (either Marine13 in Fig. **A** and **B** or ‘New Marine’ in Fig. **C**). Blue band = terrestrial calibration curve (SHCal13).

There is, however, an obvious problem with the application of these Δ*R* values; we need to know fairly precisely the terrestrial age of the context to apply the correct Δ*R* to the marine calibration. Ideally, a regional curve should be developed from measured data from around NZ, but insufficient research has been carried out in these waters. Despite the absence of a detailed curve there is sufficient detail in the Δ*R* offset to identify a significant reservoir shift between 550 and 600 cal BP (AD 1350 and 1400) which has not previously been documented. A major wiggle in the terrestrial calibration curve is present at this time. The global marine curve displays a much attenuated wiggle, and the ~ 400 ^14^C year difference between these two curves remains (Fig. 3a,b). The extreme negative Δ*R* indicated in Fig. 2 suggests, however, that this difference is actually less than 100 years at this time in this region (see for example the terrestrial and marine curves in Fig. 3c).

This has major implications for dating early sites. But, rather than being a reason not to use shell for dating, this offset could potentially enable greater refinement of site chronologies than presently possible. An example of this is given in Fig. 3, which shows paired terrestrial/marine dates from Layer 9 at Cross Creek, an early site in the Coromandel Peninsula, variously calibrated using a modern pre-AD 1950 Δ*R* of − 7 ± 45 ^14^C years (Fig. 3a), the average archaeological Δ*R* of − 15 ± 24 ^14^C years for the period between 650 and 600 cal BP (Fig. 3b), or the New Marine curve derived from the raw black coral data (Fig. 3c). Calibration using the New Marine curve (Fig. 3c) gives an age for Layer 9 of AD 1241–1329 (95.4% prob.), a result equivalent to the Kaharoa tephra—an important chronostratigraphic marker thought to immediately postdate human settlement of NZ. This tephra has been precisely dated to 1314 ± 6 AD (1310–1320 AD 68%)^44^. Table 1 presents the statistical probability for the deposition of the Kaharoa tephra at different times within the Cross Creek stratigraphy and the impact of different marine correction methods on the findings. Although the results are not conclusive, the new black coral and archaeological Δ*R* values (− 42 ± 44 and − 15 ± 24 ^14^C years (Table S2), and the New Marine curve, support deposition of the tephra between layers 5 and 7, or between layers 7 and 9, rather than before initial occupation at the site (i.e., before Layer 9) (Fig. S2 gives an extended model for layers 7 through to 9. The OxCal code is presented in Text S4).

**Table 1:** Statistical evaluation^#^ of the placement of the Kaharoa Tepha at different points of the Cross Creek sequence.

Placement of Kaharoa Tephra^	Reservoir correction method used			
	Δ*R* = − 7 ± 45 ^14^C years** (derived from modern pre-AD 1950 shells)	Δ*R* = − 42 ± 44 ^14^C years ** (derived from black coral dataset; Table S2)	Δ*R* = − 15 ± 24 ^14^C years** (derived from archaeological dataset; Table S2)	New marine curve
Before layer 9	P = 21	P = 8	P = 13	P = 3
	A_model_ = 107.5	A_model_ = 136.1	A_model_ = 95.8	A_model_ = 158
Between layers 7 and 9*	P = 26	P = 26	P = 26	P = 20
	A_model_ = 106.3	A_model_ = 136.8	A_model_ = 95.4	A_model_ = 156.9
After layer 7*	P = 13	P = 28	P = 17	P = 46
	A_model_ = 107.6	A_model_ = 134.9	A_model_ = 96.2	A_model_ = 158.7

^#^P = probability that this determination occupies that position as determined using the Outlier () function in OxCal with no parameters set (this enables the program to calculate the probability that a sample is at a particular point in the sequence)^46^ (OxCal code given in Text S4).

^The calendar age of the Kaharoa Tephra is modelled at 2σ resolution within these models (i.e., 1314 ± 12 AD).

*Layers 8 and 6 are non-cultural deposits. Layer 8 is considered by Furey et al.^45^ to be the Kaharoa Tephra.

**Calibrated using Marine13^14^ with specified Δ*R* correction applied.

These findings are of specific interest to a debate about the origins of a tephra sandwiched between layers 7 and 9. Furey et al.^45^ have previously suggested the culturally sterile yellow “sand” layer was the Kaharoa Tephra. Jacomb et al. (p. 29)^3^, however has described this association as “unconvincing” and considers this tephra layer to be redeposited. Rat gnawed seeds found within the Kaharoa Tephra at Te Rerenga in the Coromandel are a definitive indication that humans had made landfall in this region by this time^47^, so the inclusion of the ash between cultural layers at Cross Creek cannot be dismissed outright. In the absence of a renewed dating program for Cross Creek, improved calendar age resolution for shell and fishbone ^14^C dates through the development of a regionally specific marine calibration curve will be crucial to solving this debate.

## Discussion

Komugabe-Dixson et al. (p. 977–978)^20^ attributed Δ*R* shifts along the Eastern Australian coastline to the variable influence of ^14^C-depleted water from equatorial waters, possibly caused by El Niño, Southern Oscillation (ENSO) variability. They attributed older waters (i.e., more positive Δ*R* values) around Tasmania to an increased influence of older sub-Antarctic waters, but did not specifically discuss causes for the negative values observed in the black coral data that start around AD 1550 (400 BP). The extreme negative Δ*R* trend we have identified between 550 and 600 cal BP is an extension of this and, at its most negative, broadly matches the date of transition from the Medieval Climate Anomaly (∼AD 700–1350; 1250–650 BP) to the Little Ice Age (∼AD 1350–1450; 650–500 BP) (Fig. 2). Using a range of proxy climate records from subtropical and extratropical sources, Goodwin et al. (p.1212)^43^ argue that there is a “relatively abrupt shift” in mean climate state after AD 1300 resulting in winder, wetter and colder conditions across NZ (associated with a movement north of the westerly wind belt that circles Antarctica combined with El Niño conditions). These climatic shifts have been linked to the apparent patterns of East Polynesian voyaging and migration^48^. Goodwin et al. (p.14719)^48^ further suggest a brief period of finer weather 100 years later—which matches the positive Δ*R* in the archaeological data at the same time—rapidly followed by a return to wet, wild conditions. Moreover, according to Goodwin et al.^48^, El Niño-like conditions prevailed up to ∼AD 1600 (350 BP). This observation is accompanied by more positive Δ*R* values. Komugabe-Dixson et al. (p. 978)^20^ suggest that weaker gyre circulation at this time may have resulted in a northward shift of cooler, older Sub-Antarctic waters from the Subtropical Front that skirts the southeastern coast of the South Island (Fig. 1). Unfortunately, the resolution of the archaeological data between AD 1650 and 1950 (300–0 BP) is limited because of the calibration wiggles in the paired terrestrial dates.

Conditions around NZ are the result of a complex interplay of many climate systems, so it is not surprising that there are anomalies with this picture. Komugabe-Dixson et al.^20^ recorded low marine ^14^C reservoir ages for black coral from the Norfolk Ridge between AD 1700 and 1950 (250–0 BP). Petchey et al.^19^ also report a negative Δ*R* (av. –49 ± 10 ^14^C years) for modern shells from Norfolk Island. They attributed this to increased absorption of atmospheric CO_2_ caused by enhanced biological activity at the intersection of warm tropical waters and cooler waters of the Tasman Sea. There is no evidence of lower Δ*R* values in the NZ archaeological data at this time, but there are few examples from the far northern tip of the North Island where the Tasman Sea has greater influence.

The short chronology for Polynesian settlement and broad calibrated age ranges for ^14^C samples have prevented the resolution of many debates in NZ archaeology. The research presented here indicates that a better understanding of the marine ^14^C reservoir will improve this picture. In particular, it is evident that the marine ^14^C signal does not always match atmospheric ^14^C production because of the complex interrelationship of climatic and oceanic ^14^C. This difference between the marine and terrestrial ^14^C reservoirs provides a means by which we can refine chronologies that have been limited by plateaus and wiggles in the terrestrial calibration curve. Ultimately, it is important that a more precise and accurate regional marine calibration curve is produced from measured (not modelled) data. In the meantime, the Δ*R* trends identified here will enable renewed insight and investigation of the chronology of Polynesian settlement and development of Māori culture.

## Supplementary information

Supplementary file1

## Data Availability

All data is provided in the supplementary information.
