# Supplementary material for: Vital evidence: Change in the marine 14C reservoir around New Zealand (Aotearoa) and implications for the timing of Polynesian settlement
Source: Sci Rep. 2020 Aug 31;10:14266. doi: 10.1038/s41598-020-70227-3 (PMC7458910; doi:10.1038/s41598-020-70227-3)
Supplement: Supplementary file 1 — Supplementary file1 [file 41598_2020_70227_MOESM1_ESM.pdf]

Vital evidence: Change in the marine ^{14}C reservoir around New Zealand (Aotearoa) and implications for the timing of Polynesian settlement.

Fiona Petchey and Magdalena M.E. Schmid

Supporting Information Appendix (SI Appendix)

1. Evaluation of archaeological sites by 50 and 100 year blocks	Pg 1-6
• Sites dating to between 100 and 300 cal BP (AD 1650 - 1850)	Pg 1
• Sites dating to between 300 and 400 cal BP (AD 1550 - 1650)	Pg 2-3
• Sites dating to between 400 and 500 cal BP (AD 1450 - 1550)	Pg 3
• Sites dating to between 500 and 600 cal BP (AD 1350 - 1450)	Pg 3-4
• Sites dating to between 600 and 650 cal BP (AD 1300 - 1350)	Pg 4-5
• Sites older than 650 cal BP (older than AD 1300)	Pg 5-6
2. Tables	
• S1: Archaeological sites with marine/terrestrial pairs	Pg 6-11
• S2: Change in Δ*R* over time as determined by black coral (Tasmania) and New Zealand archaeological pairs	Pg 12
3. Figures	
• S1: Sites dating to <300 cal BP.	Pg 2
• S2: Calibrated results for Cross Creek, layers 7-9.	Pg 13
4. OxCal code for Cross Creek	Pg 14
5. Supporting references	Pg 15-

1. Evaluation of archaeological sites by 50 and 100 year blocks

Sites dating to between 100 and 300 cal BP (AD 1650 - AD 1850)

Ten archaeological Δ*R* pairs have mean calibrated ages dating between 100 and 300 BP. These include; Waioneke, Aotea Harbour Site 210, Hurumoimoi, two Puriri-91 sites (885 and 340), Ponui Island Site 95, Stingray Pa, Aotea Island Site 171A and Site 171B, and Aotea Island Site 16C (Figure 1, Table S1). The age of each context is difficult to assess here because of multiple wiggles in the terrestrial calibration curve. Only two of these contexts, both with mean calibrated ages greater than 200 BP, are included in Fig. 2; Aotea Site 16C (Δ*R* = 140 ± 50 ^{14}C years) and Site 171B (Δ*R* = 29 ± 39 ^{14}C years). The large offset between charcoal and shell dates from Site 16C was thought, in the original publication[1], to be caused by post-depositional disturbance. The calculated Δ*R* is, however, similar to black coral values between 200 and 300 cal BP.

The calibrated distributions for most paired shell and charcoal dates overlap when an average Δ*R* of 46 ± 49 ^{14}C years, derived from the black coral (Table S2), is used to calibrate shell dates from these young contexts (Fig. S1). Material from Ponui Site 95 Layer B, is the only exception. Schmidt (p.138)[1] suggested that this midden site, which was composed entirely of one shellfish species (*Austrovenus* sp.), was unusual for Ponui Island. Anomalous old shell dates from the lower Layer D at the same site (Table S1; sites dating to between 500 and 600 cal BP) are clear evidence of intermixing with secondary beach deposits.

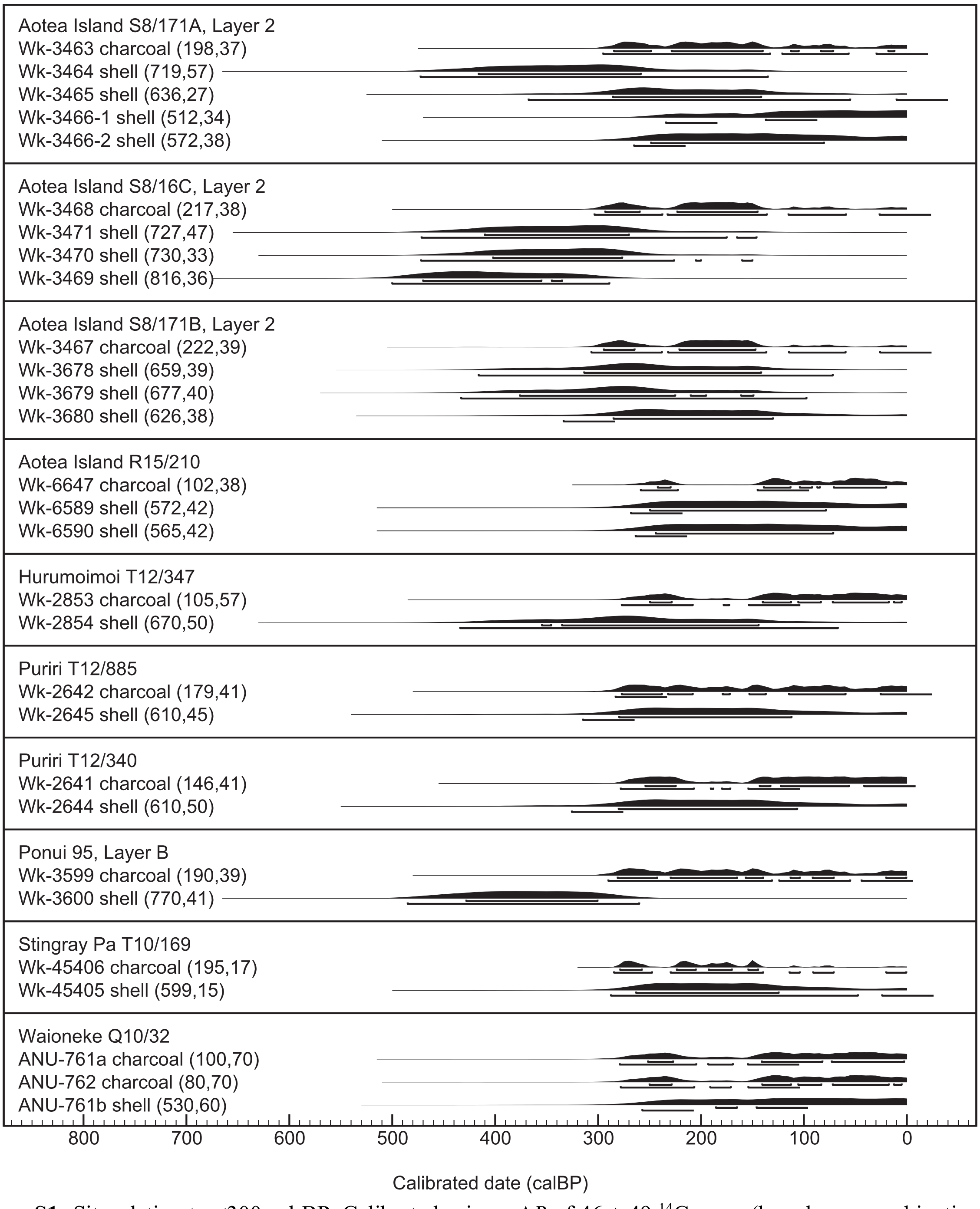

Figure S1: Sites dating to <300 cal BP. Calibrated using a ΔR of 46 ± 49 ^{14}C years (based on a combination of black coral and archaeological ΔR-values given in Table S2 for this time period).

Sites dating to between 300 and 400 cal BP (AD 1550 - AD 1650)

Delta-R (ΔR) values from ten different contexts have been identified with mean calibrated ^{14}C ages between 300 and 400 BP; Cryers Road, Aotea Harbour Site 111, Kokohuia Layers 2 and 3, Sawpit Point Layer 1B, Taputaputea middens E2 and F, Ligar Bay Driveway, Ponui Island Site 333 (Layer B), and Tata Beach Layer 3 (Fig. 1, Table S1). Between 300 and 350 cal BP the average ΔR is -26 ± 73 ^{14}C years, with a similar value between 350 and 400 cal BP (-19 ± 40 ^{14}C years) (this excludes Ponui Island Site 333 Layer B, as discussed below). Between 300 and 350 cal BP the black coral data has an average ΔR-value of 21 ± 104 ^{14}C

years with considerable variability (Table S2). Between 350 and 400 cal BP there is little change in the black coral offset with an average Δ*R* of -11 ± 35 ^{14}C years.

The Δ*R* for Ponui Island Site 333 (Layer B) is a major outlier, returning a value of -204 ± 41 ^{14}C years. Schmidt (p.134)[1] could not find any specific cause for this apparent offset. There is a single black coral date at 345 cal BP with a Δ*R* of -134 ± 40 ^{14}C years. This may hint at a short-lived event – akin to wiggles in the terrestrial calibration curve – but this needs to be investigated further.

Sites dating to between 400 and 500 cal BP (AD 1450 - AD 1550)

Eleven separate contexts have been identified with suitable marine and terrestrial pairs in this period; Tumbledown Bay Layer 3, Sawpit Point Layer 1C, Torpedo Bay Layer 3, Omaha 042, Ponui Island Site 27 (Layer D) and Site 333 (Layer D), Ligar Bay middens 1 and 2a, Kokohuia Layers 4 and 5, and Pleasant River Area 3/7 (Table S1). Between 400 and 500 cal BP the average Δ*R*-value for the archaeological dataset is -34 ± 65 ^{14}C years (Table S2). The black coral displays greater variability with average Δ*R* of -4 ± 97 ^{14}C years and -56 ± 53 ^{14}C years at 400-450 cal BP and 450-500 cal BP respectively.

Two sites hint at increased Δ*R* variability across this period. Highly negative Δ*R* values have been identified at Tumbledown Bay (-130 ± 50 ^{14}C years) and Omaha 042 (-159 ± 65 ^{14}C years). Calibrated terrestrial results for both of these contexts overlap, at 68.2% probability, a single black coral sample with a similar Δ*R*-value of -134 ± 40 ^{14}C years. Unfortunately, there is limited published contextual information for the dates from Omaha 042, but they were selected by the original researchers specifically to test the reservoir offset[2]. Archaeological deposits at Tumbledown Bay are interesting because they cover a period of major economic change whereby moa is readily available in Layer 3 (16th century AD) but absent in Layer 2 (mid 15th century AD). Artefacts recovered from the site are clear precursors to some later styles, but there is a conspicuous absence of many early styles[3,4]. Unfortunately, precise temporal control of both deposits is affected by a large wiggle in the terrestrial calibration curve resulting in multiple dates. On the basis of the present evidence it is therefore possible that Tumbledown Bay Layer 3 and Omaha 042 could belong to the next Δ*R* phase (i.e., sites dating to between 500 and 600 cal BP). A return to positive values by 450 cal BP, evident in both the archaeological and black coral data, confirms that this negative trend is of short duration.

Sites dating to between 500 and 600 cal BP (AD 1350 - AD 1450)

Between 500 and 550 cal BP the trend towards a more negative Δ*R*-value is evident in the black coral data (average Δ*R* = -61 ± 96 ^{14}C years). This trend is not immediately obvious in the archaeological values, which instead show a brief increase in Δ*R* around 500 cal BP (Fig. 2). Archaeological sites that have a mean calibrated age later than 550 BP include; Watsons Beach Area A, Pleasant River Area 1 (Layer 2), Taputaputea Area 4 (Layer Di), and Rotokura Layer 4 (Table S1). These four contexts combine to give a positive Δ*R*-value (40 ± 39 ^{14}C years; Table S2).

Eight contexts fall in the period between 550 and 600 cal BP; Pleasant River Area 7 (Layer 2a), Pouni Island Site 95 Layer D, Pleasant River Area D (layers 3 and 4), Ponui Site 14 (Layer D), Houhora, and Cooks Cove (Layer 5b) (Fig. 1, Table S1). Two additional contexts with mean calibrated ages that place them just after 600 cal BP are also included in this period; Aotea Harbour sites 218 and 433. Ponui Island Site 95 Layer D is excluded because of the inclusion of beach ridge material, as discussed above. Of the remaining contexts, Pleasant River Area D (Layer 3), Pleasant River Area 7 (Layer 2a), both Aotea Harbour sites, Pouni Island Site 14 (Layer D), and Cooks Cove Layer 5b have very negative Δ*R* values (-236 ± 55, -153 ± 92, -84 ± 26, -129 ± 121, -162 ± 45, -331 ± 56, and -287 ± 41 ^{14}C years respectively).

The calculated Δ*R* from Pleasant River Area D Layer 4 (-64 ± 206 ^{14}C years) is excluded due to complications as discussed below. A very negative Δ*R* is also evident from the single black coral date at this time (-198 ± 50 ^{14}C years at 554 cal BP [AD 1396]) (Table S2).

Variability in shell ^{14}C dates from Pleasant River, in particular Area D, has been widely discussed (see Anderson (p. 782)[5]) and previous Δ*R* research undertaken on material from Area D was specifically intended to investigate shell ^{14}C results that appeared to be too young. Based on moa eggshell and charcoal ^{14}C dates Higham (p. 152)[6] interpreted Layer 4 as a single undisturbed chronostratigraphic unit. Variable shell dates were thought to be caused by the differential impact of environmental carbon on each shell taxa. Subsequent excavation, dating and interpretation of the Pleasant River site did not clarify this issue. Smith (p. 45)[7] described a new suite of charcoal and shell ^{14}C dates from newly excavated areas (Areas 1-7) which fell into three temporal groups; 14th, and 14th to early 15th, and late 15th - 16th centuries AD, but he noted that the ages were not always in chrono-stratigraphic agreement within the same excavation unit and layer. Ultimately, Smith (p. 48)[7] concluded that the earlier Area D excavation represented a mixing of material from separate occupations along the eroding river edge. Subsequent dating of four marine dates (*Thyrsites atun* [snapper] gelatin and *Austrovenus stutchburyi*) from Area 7, Layer 2a gave consistent results, but indicated a significant marine offset (average Δ*R* of -236 ± 55 ^{14}C years) comparable to the Area D Layer 3 Δ*R*-value of -153 ± 92 ^{14}C years. Our findings suggest that the variability in the shell dates from Pleasant River may partly have been caused by rapid change in the marine Δ*R* over a relatively short period of time (probably less than 50 years) just before 600 cal BP. We have not included the Δ*R* for Pleasant River Layer 4 in the statistical evaluation (Table S2), because considerable variation in the marine values skew the average value (two *Paphies australis*, an *Austrovenus stutchburyi* and a *Cookia sulcata* date; the conventional ^{14}C ages of which range from 740 ± 45 to 1120 ± 45 ^{14}C years resulting in Δ*R* between 119 ± 61 and -261 ± 61 ^{14}C years) (see Table S1). It seems unlikely that a rapid change in marine reservoir offset could be responsible for this observed degree of offset within a single layer, and that Smith's conclusion of post-depositional mixing is correct, but the possibility is worth further investigation.

The chronologies of several other sites/contexts in this time range have also been interpreted as problematic. The Cooks Cove paired dates from Layer 5b were noted by the excavators to only just overlap at "2 sigma"[8]. The reservoir offset (Δ*R* = -162 ± 45 ^{14}C years; Table S1) is, however, compatible with the extreme negative Δ*R* trend identified at this time (Fig. 2). Aotea Harbour sites 433 and 218 were specifically sampled for Δ*R* research. McKinnon (p. 85, 87)[9] considered Δ*R* from both sites to be abnormal, and probably the result of inbuilt age in the charcoal. The extreme negative values obtained also fit with observations from other sites at this time.

Sites dating to between 600 and 650 cal BP (AD 1300 - AD 1350)

Many of the contexts that date around 600 BP can be placed in either this, or the earlier 550-600 cal BP time frame. We have assumed the majority of contexts included in this section date prior to the major offset in Δ*R* identified between 550 and 600 cal BP based on a combination of the terrestrial calibrated age range and a more positive Δ*R* offset. Taputaputea Area 6 (Δ*R* = -44 ± 42 ^{14}C years), Shag River Mouth Dune Layer 4 (Δ*R* = -24 ± 58 ^{14}C years), Layer 7 (Δ*R* = -2 ± 52 ^{14}C years) and Layer 11 (Δ*R* = -60 ± 64 ^{14}C years), Cabana Lodge (Δ*R* = -4 ± 40 ^{14}C years), Wairau Bar midden (Δ*R* = 77 ± 37 ^{14}C years), Watsons Beach Layer 2 (2 ± 33 ^{14}C years) and Cross Creek (Δ*R* = -5 ± 60 ^{14}C years) all date within this period (Table S1). Overall, these sites display relatively little variation in Δ*R* and return an average value of -15 ± 24 ^{14}C yrs (excludes Wairau Bar; see discussion below). There are no black coral values from this period.

Shag River Mouth is one of the most extensively dated archaeological sites in NZ[6,10-12]. The site is thought to have been occupied for between 20 and 50 years in the 14th century AD. This conclusion was based on a combination of shell (n=15; AD 1329-1373 (68% prob.)), charcoal (n=14; AD 1330-1346 and AD 1393-1409) and eggshell dates (n=3; AD 1335-1336 and AD 1405-1433)[10]. Following the initial dating of the site, a number of dates[12] were obtained of various bird taxa (and *Rattus exulans,* which are not reported here). The list in Table S1 includes terrestrial and marine animal bones from this study. However, the Chi square statistic for the terrestrial dates from Layer 4 (charcoal, moa bone gelatin and pigeon ultrafiltered gelatin) indicates these dates are variable ($\chi^2_{4:0.05}$=10.74<9.49). Higham et al. (p. 371)[12] also noted a significant difference between dates on gelatin and ultrafiltered gelatin measured on the same pigeon bone (OxA-13239). They concluded the ultrafiltrated bone gelatin date, while younger, was more reliable. To test this assumption FP compared dates on gelatin (Wk-5345a) and ultrafiltrated gelatin (Wk-5345b) from the same barracouta (*Thyrsites atun*) bone at the Waikato Radiocarbon Dating Laboratory but found no evidence of contamination. Because of uncertainties with bone pretreatment we have excluded the OxA dates from further analysis.

The terrestrial age used here for Wairau Bar is based on twelve moa eggshell samples from an oven, excavated in 2009, considered to represent a single cooking and discard event. The four shell dates presented in Table S1 come from the "Layer 4 midden" and "main occupation" layers from earlier excavations at the site. Consequently, the shell/terrestrial pairs do not conform to strict Δ*R* guidelines (see methodology section). Our inclusion of these separate contexts in Table S1 assumes deposition close in time, as suggested by Walter et al. (p. 8)[13] who interpreted the site as a large village that was occupied for decades around AD 1320–1350 (68% prob.) based on the aforementioned eggshell dates[14] and limited stratigraphic complexity across the site. In this scenario, the nearby burial ground, also dated using multiple moa eggshell samples, is considered to be of a similar age to the oven[15]. Assuming an association between the midden/main occupation and the oven is correct, a return to a much more positive Δ*R* is evident (77 ± 37 ^{14}C years). However, if the shell dates come from midden deposits that are slightly older than the single use oven, the calculated Δ*R* would be elevated. Delta-R values from other sites in this time period, while not statistically different to the Δ*R* from Wairau Bar, do tend to be more negative (Table S1, Fig. 2). Unfortunately, there are no black coral dates around 600 cal BP for comparison. Because of uncertainties about the contemporaneity of oven and midden deposits, we have removed Wairau Bar from the temporal average (Table S2). Multiple moa eggshell dates and ultrafiltered moa collagen ^{14}C dates from Wairau Bar have also been published separately[16] but without specific context information. They are, therefore, excluded from our analysis.

Sites older than 650 cal BP (older than AD 1300)

Shell/charcoal pairs from Fyffes site return a Δ*R* of -2 ± 42 ^{14}C years (Table S1 and Table S2). This matches the single black coral Δ*R* at this time (-3 ± 36 ^{14}C years). The calibrated ages of charcoal and shell samples from this site suggest an early to mid 13th century AD occupation and have been considered to be too old given orthodox opinions of first settlement age. McFadgen[17] concluded that both natural and cultural materials had become mixed during the formation of a beach ridge. He further suggested the site represented a transient settlement most likely dating to 500 cal BP. This was not endorsed by Anderson (p. 126)[18] who suggested a more permanent settlement was likely based on the extent of the site and proximity to a nearby Archaic cemetery. Instead, Anderson (p. 777) [5] concluded that local upwelling and/or ancient carbon derived from nearby limestone outcrops might account for the old shell dates. He also excluded a single unidentified charcoal date because of the possibility of inbuilt age (ibid:782; we note however, that the charcoal was identified to *Leptospermum* and *Coprosma* species. Both could theoretically have up to 100 years of inbuilt age[19]). Higham (p. 137-8) [6], using

modern shellfish from this location, tested the possibility that ancient ^{14}C derived from upwelled waters or limestone could have affected the shell ages, but did not find a significant offset. He suggested seasonal variation in upwelled water along the Subtropical Front (Fig. 1) could account for the older than expected ages. Intra-shell radiocarbon studies of marine molluscs from California[20,21] have demonstrated that this is possible. Studies along the NZ coastline, however, suggest a long and sustained period of upwelling would be required in order to influence near-shore animals in this region[22].

Moa eggshell dates from Fyffes are also reported in Holdaway et al.[16]. These range from 592 ± 17 ^{14}C years to 683 ± 15 ^{14}C years, with one outlier of 1082 ± 15 ^{14}C years. This range of ages could indicate mixing of subfossil and archaeological material. Unfortunately, no information on sample provenance is published and therefore these dates are not included in Table S1. Ultimately, the exact age of Fyffes site remains problematic, but the calibrated age ranges for the dates given in Table S1 (AD 1075-1266 [875-684 cal BP] at 68% prob.; AD 1190-1372 [760-579 cal BP] at 68% prob.) overlap orthodox opinions of early settlement and cannot presently be excluded on the basis of parameters used in this study. Combined, the shell and charcoal dates support an early settlement age for the site.

2. Table S1: Archaeological sites with marine/terrestrial Δ*R* pairs.

Site	Context	Details#	Material	Terrestrial statistics median age (68% prob. range)**	Δ*R* ^{14}C years (68% prob.)	Δ*R* statistics^{+}	Reference/Comments
**Sites dating to between 100 and 300 cal BP (AD 1650 - 1850)**							
**Waioneke**<br>NZAA # = Q10/32<br>Imperial # = N37/25	Large pit	**ANU-761a; 100±70**<br>**ANU-762; 80±70**<br>ANU-761b; 530±60	**Charcoal: identified**<br>**Charcoal: identified**<br>Unidentified shell	<200 BP<br>$\chi^2_{1:0.05}$=0.04<3.84		-	NZ Radiocarbon Database: https://www.waikato.ac.nz/waikato/nzcd/index.html<br>Sealed by debris from final period of occupation.
**Aotea Harbour (South Head)**<br>NZAA # = R15/210<br>Imperial # = N73/277	Midden	**Wk-6647; 102±38**<br>Wk-6589; 572±42<br>Wk-6590; 565±42	**Charcoal identified**<br>*Paphies subtriangulata*<br>*Paphies australis*	<200 BP	-	-	McKinnon (p. 88)[9]
**Hurumoimoi**<br>NZAA # = T12/347<br>Imperial # = N49/379	Hangi, Layer 3	**Wk-2853; 105±57***<br>Wk-2854; 670±50	**Charcoal: identified**<br>*Paphies australis*	<200 BP	-	-	Doelman[23]
**Puriri Site 91**<br>NZAA # = T12/340<br>Imperial # = N49/372	Area D2, Phase II midden	**Wk-2641: 146±41***<br>Wk-2644; 610±50	**Charcoal: identified**<br>*Austrovenus stutchburyi*	<200 BP	-	-	Bedford and Allen (p. 131)[24]
**Puriri Site 91**<br>NZAA # = T12/885	Assemblage 23b.	**Wk-2642; 179±41***<br>Wk-2645; 610±45*	**Charcoal: identified**<br>*Austrovenus stutchburyi*	<200 BP	-	-	Bedford and Allen (p. 131)[24]
**Ponui Island Site 95**	Layer B	**Wk-3599; 190±39**<br>Wk-3600; 770±41	**Charcoal: identified**<br>*Austrovenus stutchburyi*	<200 BP	-	-	Schmidt (p. 138)[1] suggested possible mixing with natural deposits
**Stingray Pa**<br>NZAA # = T10/169<br>Imperial # = N40/11	Pit A	**Wk-45406 195±17**<br>Wk-45405 599±15	**Charcoal: identified**<br>*Austrovenus stutchburyi*	<200 BP	-	-	Furey et al.[25]
**Aotea Island 171A**<br>NZAA # = S8/171A<br>Imperial # = N30/326	Layer 2	**Wk-3463; 198±37***<br>Wk-3464; 719±57<br>Wk-3465; 636±27<br>Wk-3466-1; 512±34<br>Wk-3466-2; 572±38	**Charcoal: identified**<br>*Paphies subtriangulata*<br>*Paphies australis*<br>*Austrovenus stutchburyi*	<200 BP	-	-	Schmidt (p.136)[1] suggested disturbed by ploughing and historic building activities
**Aotea Island 171B**<br>NZAA # = S8/171 B<br>Imperial # = N30/326	Layer 2	**Wk-3467; 222±39**<br>Wk-3678; 659±39<br>Wk-3679; 677±40<br>Wk-3680; 626±38	**Charcoal: identified**<br>*Paphies subtriangulata*<br>*Paphies australis*<br>*Austrovenus stutchburyi*	195 cal BP<br>(147-290 BP)	34±66<br>53±67<br>2±66	29±39<br>$\chi^2_{2:0.05}$=0.30<5.99<br>gSD=26	Schmidt (p. 124)[1]
**Aotea Island 16C**<br>NZAA # = S8/16C<br>Imperial # = N30/59	Layer 2	**Wk-3468; 217±38**<br>Wk-3471; 727±47<br>Wk-3470; 730±33<br>Wk-3469; 816±36	**Charcoal: identified**<br>*Paphies subtriangulata*<br>*Paphies australis*<br>*Austrovenus stutchburyi*	192 cal BP<br>(147-289 BP)	110±72<br>110±62<br>196±64	140±38<br>$\chi^2_{2:0.05}$=1.17<5.99<br>gSD=50	Schmidt (p. 136)[1] suggested post-depositional disturbance

Table S1 cont.

Site	Context	Details#	Material	Terrestrial statistics median age (68% prob. range)**	ΔR ^{14}C years (68% prob.)	ΔR statistics	Reference/Comments
**Sites dating to between 300 and 400 cal BP (AD 1550 - 1650)**							
**Cryers Road**<br>NZAA # = R11/1519	Area 41 Base of shell deposit	**Wk-1126; 300±50**<br>Wk-1133; 690±45	**Charcoal: identified**<br>*Austrovenus stutchburyi*	327 cal BP (282-442BP)	-26±73	-	Fredericksen and Visser[26]
**Aotea Harbour (South Head)**<br>NZAA # = R15/111<br>Imperial # = N73/1	Midden	**Wk-6650; 323±60**<br>Wk-6666; 589±42<br>Wk-6667; 672±42	**Charcoal: identified**<br>*Austrovenus stutchburyi*<br>*Paphies australis*	367 cal BP (292-447BP)	-24±88<br>58±88	17±63<br>$\chi^2_{1:0.05}$=0.43<3.84;<br>gSD=58	McKinnon (p. 89)[9]
**Kokohuia**<br>NZAA # = O06/317<br>Imperial # = N18/232	Layer 2	**Wk-3695; 330±43**<br>**Wk-4484; 379±42**<br>**Wk-4485; 342±41**<br>**Wk-4486; 408±41**<br>**Wk-4487; 380±40**<br>Wk-2564; 760±60<br>Wk-3696; 710±41<br>Wk-3697; 765±40<br>Wk-3698; 677±38<br>Wk-3699; 764±41<br>Wk-3701; 802±41	**Charcoal: identified**<br>**Charcoal: identified**<br>**Charcoal: identified**<br>**Charcoal: identified**<br>**Charcoal: identified**<br>*Austrovenus stutchburyi*<br>*Paphies australis*<br>*Austrovenus stutchburyi*<br>*Venerupis largillierti*<br>*Turbo smaragda*<br>*Perna canaliculus*	388 cal BP (331-452BP)<br>$\chi^2_{4:0.05}$=2.29<9.49	-8±76<br>-57±62<br>-2±62<br>-89±60<br>-3±62<br>35±62	-22±26<br>$\chi^2_{5:0.05}$=2.64<11.07<br>gSD=45	Schmidt (p. 117)[1]
**Sawpit point**<br>NZAA # = N26/214	Layer 1B	**Wk-4028; 346±40**<br>Wk-4030; 788±37<br>Wk-4033; 826±41	**Charcoal: identified**<br>*Austrovenus stutchburyi*<br>*Paphies australis*	389 cal BP (315-445BP)	38 ± 60<br>75 ± 62	56±44<br>$\chi^2_{1:0.05}$=0.18<3.84<br>gSD=26	Schmidt (p. 133)[1]
**Taputapuatea**<br>NZAA # = T11/914	Midden F	**Wk-43231; 354±20**<br>Wk-41171; 781±30	**Charcoal: identified**<br>*Paphies australis*	392 cal BP (324-441BP)	30 ± 44	-	Hoffmann (p. 30)[27]
**Taputapuatea**<br>NZAA # = T11/914	Midden E2	**Wk-43291; 378±20**<br>Wk-41169; 772±26	**Charcoal: identified**<br>*Paphies australis*	387 cal BP (328-455BP)	1 ± 52	-	Hoffmann (p. 30)[27]
**Ligar Bay Driveway**<br>NZAA # = N25/26<br>Imperial # = S8/40	Layer 4	**Wk-3533; 387±39**<br>Wk-3534; 737±34<br>Wk-3535; 761±34<br>Wk-3536; 748±39<br>Wk-3537; 756±38<br>Wk-3538; 704±39	**Charcoal: identified**<br>*Paphies australis*<br>*Paphies australis*<br>*Austrovenus stutchburyi*<br>*Turbo smaragda*<br>*Cominella adspersa*	386 cal BP (325-458BP)	-55±64<br>-31±64<br>-44±66<br>-35±66<br>-88±66	-50±30<br>$\chi^2_{4:0.05}$=0.49<9.49<br>gSD=23	Schmidt (p. 133)[1]
**Ponui Island**<br>Imperial # = N43/333<br>NZAA # = S11/340	Layer B	**Wk-3582; 399±42**<br>Wk-3583; 590±41<br>Wk-3584; 523±40<br>Wk-3585; 517±39	**Charcoal: identified**<br>*Paphies australis*<br>*Austrovenus stutchburyi*<br>*Saccostrea glomerata*	397 cal BP (327-487BP)	-157 ± 63<br>-224 ± 62<br>-230 ± 62	-204±37<br>$\chi^2_{2:0.05}$=0.84<5.99<br>gSD=41	Schmidt (p. 134)[1] suggested charcoal inbuilt age, misidentification or long storage.
**Kokohuia**<br>NZAA # = O06/317<br>Imperial # = N18/232	Layer 3	**Wk-3703; 407±40**<br>**Wk-4540; 357±40**<br>**Wk-4541; 352±42**<br>Wk-3704; 705±40<br>Wk-3705; 753±42<br>Wk-3706; 719±42<br>Wk-3707; 750±38<br>Wk-3709; 736±37	**Charcoal: identified**<br>**Charcoal: identified**<br>**Charcoal: identified**<br>*Paphies australis*<br>*Austrovenus stutchburyi*<br>*Venerupis largillierti*<br>*Turbo smaragda*<br>*Perna canaliculus*	385 cal BP (325-451BP)<br>$\chi^2_{3:0.05}$=1.13<5.99	-76±64<br>-28±64<br>-62±64<br>-32±62<br>-46±62	-49±29<br>$\chi^2_{4:0.05}$=0.4<9.49<br>gSD=20	Schmidt (p. 117)[1]
**Tata Beach**	Layer 3	**Wk-4893; 418±36**<br>**Wk-4894; 357±40**<br>Wk-4864; 784±40<br>Wk-4865; 807±35<br>Wk-4866; 837±40<br>Wk-4867; 734±40<br>Wk-5134; 675±39<br>Wk-5135; 684±47<br>Wk-6032; 780±44<br>Wk-6033; 746±46<br>Wk-6034; 715±42	**Charcoal: identified**<br>**Charcoal: identified**<br>*Paphies australis*<br>*Austrovenus stutchburyi*<br>*Paphies australis*<br>*Austrovenus stutchburyi*<br>*Thyrsites atun*<br>*Pseudophycis bachus*<br>*Pseudophycis bachus*<br>*Thyrsites atun*<br>*Pseudophycis bachus*	402 cal BP (330-489 BP)<br>$\chi^2_{1:0.05}$=1.28<3.84	-12 ± 68<br>10 ± 66<br>40 ± 68<br>-62 ± 68<br>-121 ± 68<br>-112 ± 72<br>-16 ± 70<br>-50 ± 72<br>-80 ± 70	-43±23<br>$\chi^2_{8:0.05}$=5.09<15.51<br>gSD=55	Petchey (p. 147)[11]

Table S1 cont.

Site	Context	Details#	Material	Terrestrial statistics median age (68% prob. range)**	Δ*R* ^{14}C years (68% prob.)	Δ*R* statistics	Reference/Comments
**Sites dating to between 400 and 500 cal BP (AD 1450 - 1550)**							
**Tumbledown Bay**<br>NZAA # = N37/12<br>Imperial # = S94/30 = imperial	Layer 3	**NZ-7656; 418±47**<br>NZ-7654; 706±50<br>NZ-7745; 686±38	**Charcoal: identified**<br>*Paphies subtriangulata*<br>*Haloitis iris*	432 cal BP<br>(328-499 BP)	-118 ± 74<br>-140 ± 66	-130±50<br>$\chi^2_{1:0.05}=0.05<3.84$<br>gSD=16	Anderson (p. 128)[18]; Allingham[13]
**Sawpit point**<br>NZAA # = N26/214	Layer 1C	**Wk-4029; 414±41**<br>Wk-4031; 804±41<br>Wk-3099; 800±50<br>(Wk-4032;1868±43)	**Charcoal: identified**<br>*Turbo smaragda*<br>*Paphies australis*	415 cal BP<br>(330-495 BP)	-20 ± 65<br>-22 ± 71	-21±49<br>$\chi^2_{1:0.05}=0.00<3.84$<br>gSD=1	Schmidt (p.140)[1]; Barber[28]<br>Note: Wk-4032 is subfossil material and is excluded from this evaluation.
**Torpedo Bay**<br>NZAA # = T11/914	Layer 3 (features 3, 5 and 31)	**Wk-31977; 398±29**<br>Wk-31976; 875±35<br>Wk-31101; 698±33<br>Wk-31978; 773±25	**Charcoal: identified**<br>*Paphies australis*<br>*Austrovenus stutchburyi*<br>*Paphies australis*	419 cal BP<br>(330-491 BP)	62 ± 58<br>-114 ± 58<br>-44 ± 53	-33±33<br>$\chi^2_{2:0.05}=4.68<5.99$<br>gSD=89	Campbell et al. [29]
**Omaha 042**<br>NZAA # = R09/887	Sample 2	**Wk-12919; 409±33**<br>Wk-11435; 662±46	**Charcoal: identified**<br>*Paphies australis*	432 cal BP<br>(332-494 BP)	-159 ± 65	-	Bickler et al. (p. 50-51, 165-166)[2]
**Ponui Island**<br>Imperial # = N43/333<br>NZAA # = S11/340	Layer D	**Wk-3586; 411±39**<br>**Wk-3587; 432±40**<br>Wk-3588; 889±40<br>Wk-3589; 926±38	**Charcoal: identified**<br>**Charcoal: identified**<br>*Paphies australis*<br>*Austrovenus stutchburyi*	459 cal BP<br>(339-495 BP)<br>$\chi^2_{1:0.05}=0.14<3.84$	54 ± 54<br>91 ± 52	73±38<br>$\chi^2_{1:0.05}=0.24<3.84$<br>gSD=26	Schmidt (p. 127, 135)[1]
**Ligar Bay**<br>NZAA # = N25/95	Midden 1	**Wk-3539; 442±38**<br>Wk-3540; 875±39<br>Wk-3541; 815±38<br>Wk-3542; 841±40<br>Wk-3543; 869±40	**Charcoal: identified**<br>*Paphies subtriangulata*<br>*Paphies australis*<br>*Austrovenus stutchburyi*<br>*Turbo smaragda*	461 cal BP<br>(338-419 BP)	25±56<br>-34±56<br>-9±57<br>19±57	0±29<br>$\chi^2_{3:0.05}=0.70<7.81$<br>gSD=27	Schmidt (p. 131)[1]
**Kokohuia**<br>NZAA # = O06/317<br>Imperial # = N18/232	Layer 4	**Wk-3711; 366±40**<br>**Wk-4482; 449±41**<br>**Wk-4483; 494±40**<br>**Wk-4542; 433±40**<br>Wk-3712; 810±40<br>Wk-3713; 750±41<br>Wk-3715; 770±39<br>Wk-3716; 754±40	**Charcoal: identified**<br>**Charcoal: identified**<br>**Charcoal: identified**<br>**Charcoal: identified**<br>*Paphies australis*<br>*Austrovenus stutchburyi*<br>*Turbo smaragda*<br>*Cominella virgata*	479 cal BP<br>(453-509 BP)<br>$\chi^2_{3:0.05}=5.27<7.81$	-32±70<br>-91±70<br>-72±69<br>-88±70	-71±35<br>$\chi^2_{3:0.05}=0.45<7.81$<br>gSD=27	Schmidt (p. 120)[1]
**Ponui Island**<br>**Site 27**	Layer D	**Wk-3596; 465±43**<br>Wk-3597; 744±40<br>Wk-3598; 821±40	**Charcoal: identified**<br>*Paphies australis*<br>*Austrovenus stutchburyi*	483 cal BP<br>(454-519 BP)	-126 ± 58<br>-50 ± 58	-88±42<br>$\chi^2_{2:0.05}=0.86<3.84$<br>gSD=54	Schmidt (p. 129)[1]
**Ligar Bay**<br>NZAA # = N25/95	Midden 2a	**Wk-3544; 466±38**<br>Wk-3545; 786±40<br>Wk-3546; 921±39<br>Wk-3547; 841±38<br>Wk-3548; 887±37<br>Wk-3549; 881±41	**Charcoal: identified**<br>*Paphies subtriangulata*<br>*Paphies australis*<br>*Austrovenus stutchburyi*<br>*Turbo smaragda*<br>Unidentified shell	488 cal BP<br>(461-516 BP)	-86±54<br>49±54<br>-31±53<br>14±52<br>10±56	-9±25<br>$\chi^2_{4:0.05}=3.67<9.49$<br>gSD=52	Schmidt (p. 131)[1]
**Kokohuia**<br>NZAA # = O06/317<br>Imperial # = N18/232	Layer 5	**Wk-3718; 384±41**<br>**Wk-3719; 443±42**<br>**Wk-3720; 492±41**<br>**Wk-3721; 505±45**<br>**Wk-4543; 484±42**<br>**Wk-4544; 407±41**<br>Wk-3722; 787±38<br>Wk-3723; 808±38<br>Wk-3724; 820±40<br>Wk-3726; 842±44<br>Wk-3727; 823±44	**Charcoal: identified**<br>**Charcoal: identified**<br>**Charcoal: identified**<br>**Charcoal: identified**<br>**Charcoal: identified**<br>**Charcoal: identified**<br>*Paphies australis*<br>*Austrovenus stutchburyi*<br>*Turbo smaragda*<br>*Cominella virgata*<br>*Perna canaliculus*	491 cal BP<br>(472-511 BP)<br>$\chi^2_{5:0.05}=6.91<11.07$	-70±63<br>-49±63<br>-37±64<br>-14±68<br>-34±68	-42±30<br>$\chi^2_{4:0.05}=0.40<9.49$<br>gSD=21	Schmidt (p. 120)[1]
**Pleasant River, Area 3/7**<br>NZAA # = J43/1<br>Imperial # = S155/2	Layer 1	**NZA-2802;494±62**<br>Wk-3508; 881±40<br>Wk-3509; 757±34	**Charcoal: identified**<br>*Austrovenus stutchburyi*<br>*Austrovenus stutchburyi*	497 cal BP<br>(453-543 BP)	-16 ± 69<br>-140 ± 66	-81±48<br>$\chi^2_{1:0.05}=1.69<3.84$<br>gSD=88	Smith (p. 41, 45-48)[7]. Adjacent to Area 7. Considered to be material derived from Layer 2a.

Table S1 cont.

Site	Context	Details#	Material	Terrestrial statistics median age (68% prob. range)**	Δ*R* ^{14}C years (68% prob.)	Δ*R* statistics	Reference/Comments
**Sites dating to between 500 and 600 cal BP (AD 1350 - 1450)**							
**Watsons Beach, Area A**<br>NZAA # = H45/10<br>Imperial # = S172/55)	Layer 2	**Wk-12962; 494±42**<br>Wk-12960; 1010±36<br>Wk-12961; 982±38	**Moa eggshell**<br>"Mussel"<br>"Mussel"	506 cal BP<br>(475-532 BP)	111 ± 51<br>82 ± 52	97±37<br>$\chi^2_{1:0.05}=0.16<3.84$<br>gSD=21	Kirk (p. 82)[30]; Jacomb and Darmody (p. 54)[31]
**Pleasant River, Area 1**<br>NZAA # = J43/1<br>Imperial # = S155/2	Layer 2 midden	**NZ-7960; 507±64**<br>Wk-2370; 970±50<br>Wk-2753; 910±45<br>Wk-2851; 970±35	**Charcoal: identified**<br>*Austrovenus stutchburyi*<br>*Austrovenus stutchburyi*<br>*Austrovenus stutchburyi*	506 cal BP<br>(466-551 BP)	63 ± 76<br>2 ± 72<br>62 ± 66	43±41<br>$\chi^2_{2:0.05}=0.48<5.99$<br>gSD=35	Smith (p. 36)[7]; Higham[6]
**Taputapuatea**<br>T11/914 = NZAA	Area 4, Layer Di, fea 61	**Wk-43227; 506±20**<br>Wk-41164; 926±21	**Charcoal: identified**<br>*Paphies australis*	511 cal BP<br>(501-520 BP)	20 ± 26	-	Hoffmann (p. 30)[27]
**Rotokura**<br>NZAA # = O27/1<br>Imperial # = S14/1	Layer 3/4	**NZ-1105; 586±57**<br>**Wk-5482; 529±44**<br>**Wk-5483; 598±34**<br>**Wk-5484; 543±35**<br>Wk-4887; 1039±38<br>Wk-4953; 936±41<br>Wk-4954; 956±43<br><br>Wk-4955; 906±42<br>Wk-4953; 936±41<br>Wk-4954; 956±43	**Charcoal: not identified**<br>**Charcoal: identified**<br>**Duplicate of Wk-5482**<br>**Duplicate of Wk-5482**<br>*Paphies australis*<br>*Protothaca crassicosta*<br>*Lunella smaragda, Haliotis iris*<br>*Pagrus auratus* (gelatin)<br>*Pagrus auratus* (gelatin)<br>*Pagrus auratus* (gelatin)	534 cal BP<br>(519-550BP)<br>$\chi^2_{3:0.05}=2.14<7.81$	92 ± 46<br>-12 ± 49<br>8 ± 50<br><br>-42 ± 50<br>-12 ± 49<br>8 ± 50	9±20<br>$\chi^2_{5:0.05}=4.66<11.07$;<br>gSD=46	Petchey (p. 145)[11]. Note: Layer 3 was sterile clay, therefore association with Layer 4 is considered most likely. Unidentified charcoal result (NZ-1105) has been included in this analysis because the result is identical to other charcoal results.
**Pleasant River, Area 7**<br>NZAA # = J43/1<br>Imperial # = S155/2	Layer 2a	**NZA-3740; 624±65**<br>**Wk-5169; 577±45**<br>Wk-3510; 719±31<br>Wk-4956; 792±41<br>Wk-5031; 676±38<br>Wk-5036; 782±41	**Charcoal: identified**<br>**Moa bone (gelatin)**<br>*Austrovenus stutchburyi*<br>*Thyrsites atun* (gelatin)<br>*Thyrsites atun* (gelatin)<br>*Thyrsites atun* (gelatin)	552 cal BP<br>(519-625 BP)<br>$\chi^2_{1:0.05}=0.35<3.84$	-255 ± 49<br>-184 ± 56<br>-300 ± 54<br>-194 ± 56	-236±27<br>$\chi^2_{3:0.05}=2.98<7.81$<br>gSD=55	Petchey (p. 89)[11]
**Ponui Island**<br>**Site 95**	Layer D	**Wk-3601;** 595±43<br>Wk-3602-1; 1558±41<br>Wk-3602-2; 1480±51	**Charcoal: identified**<br>*Austrovenus stutchburyi*<br>*Austrovenus stutchburyi*	553 cal BP<br>(525-625 BP)	580 ± 58<br>500 ± 66	545±43<br>$\chi^2_{1:0.05}=0.83<3.84$<br>gSD=57	Schmidt (p. 138)[1] suggested mixing with natural deposits.
**Pleasant River, Area D**<br>NZAA # = J43/1<br>Imperial # = S155/2	Layer 4	**Wk-2741; 650±45**<br>**Wk-2758; 650±45**<br>**Wk-2759; 590±40**<br>**Wk-2760; 600±45**<br>**Wk-2789; 550±90**<br>Wk-2761; 1120±45<br>Wk-2762; 1110±45<br>Wk-2771; 740±45<br>Wk-2772; 780±45	**Moa eggshell**<br>**Moa eggshell**<br>**Moa eggshell**<br>**Moa eggshell**<br>**Charcoal: identified**<br>*Austrovenus stutchburyi*<br>*Paphies australis*<br>*Cookia sulcata*<br>*Paphies australis*	569 cal BP<br>(492-637 BP)<br>$\chi^2_{4:0.05}=2.23<9.49$	119 ± 61<br>109 ± 61<br>-261 ± 61<br>-221 ± 61	-64±31<br>$\chi^2_{3:0.05}=34.10<7.81$<br>gSD=206	Smith (p. 48)[7] suggested post-depositional mixing in Area D. Higham (p. 141-144)[6] considered this unlikely, instead suggesting dietary/environmental offsets in shell.
**Pleasant River, Area D**<br>NZAA # = J43/1<br>Imperial # = S155/2	Layer 3	**Wk-2765; 600±45**<br>**Wk-2790; 700±90**<br>Wk-2763; 920±45<br>Wk-2764; 790±45	**Moa eggshell**<br>**Charcoal: identified**<br>*Austrovenus stutchburyi*<br>*Paphies australis*	584 cal BP<br>(507-643 BP)<br>$\chi^2_{1:0.05}=0.99<3.84$	-88 ± 76<br>-218 ± 76	-153±54<br>$\chi^2_{1:0.05}=1.46<3.84$<br>gSD=92	Smith (p. 48)[7] suggested post-depositional mixing in Area D. Higham (1993:141-144) considered this unlikely, instead suggesting dietary/environmental offsets in shell.
**Ponui Island**<br>**Site 14**	Layer D	**Wk-3590; 621±42**<br>Wk-3591; 924±43<br>Wk-3592; 939±39<br>Wk-3593; 911±39<br>Wk-3594-1; 899±40<br>Wk-3594-2; 932±40	**Charcoal: identified**<br>*Paphies australis*<br>*Austrovenus stutchburyi*<br>*Perna canaliculus*<br>*Saccostrea glomerata*	591 cal BP<br>(541-630 BP)	-80 ± 58<br>-66 ± 56<br>-94 ± 56<br>-106 ± 56<br>-72 ± 56	-84±26<br>$\chi^2_{4:0.05}=0.34<9.49$<br>gSD=16	Schmidt (p. 127)[1]
**Houhora (Mt Camel)**<br>NZAA # = N03/59<br>Imperial # = **N6/4**	Layer 2b	**NZA-2436; 632±86**<br>**NZA-2437; 774±87**<br>NZ-7920; 812±37<br><br>Wk-5034; 963±41<br>Wk-5035; 1052±42	**Charcoal: identified**<br>**Charcoal: identified**<br>*Austrovenus stutchburyi,*<br>*Paphies australis*<br>*Lunella smaragda*<br>*Austrovenus stutchburyi*	592 cal BP<br>(534-649 BP)<br>$\chi^2_{1:0.05}=1.35<3.84$	-256 ± 90<br><br>-106 ± 90<br>-17 ± 93	-129±53<br>$\chi^2_{2:0.05}=3.51<5.99$<br>gSD=121	Petchey (p. 140)[32]; Furey (p. 35)[33].<br>Note: NZA-2437 and NZ-7920 taken from latex pull with possibility of retouching.
**Cooks Cove**<br>NZAA # = Z17/311<br>Imperial # = N90/644	Layer 5b	**Wk-23490; 624±30**<br><br>Wk-23489; 844±33	**Moa bone (ultrafiltered gelatin)**<br>*Haloitis iris*	597 cal BP<br>(544-629 BP)	-162±45	-	Walter et al. (p. 15-17)[34]
**Aotea Harbour 433 (South Head)**<br>NZAA # = R15/433	Midden	**Wk-6649; 693±44**<br>Wk-6660; 664±40<br>Wk-6661; 752±41<br>Wk-6662; 770±41	**Charcoal: identified**<br>*Austrovenus stutchburyi*<br>*Paphies subtriangulata*<br>*Paphies australis*	602 cal BP<br>(561-654 BP)	-395±58<br>-308±58<br>-290±58	-331±34<br>$\chi^2_{2:0.05}=1.87<5.99$<br>gSD=56	McKinnon (p. 85)[9] suggested inbuilt age in the charcoal
**Aotea Harbour 218 (South Head)**<br>NZAA # = R15/218<br>Imperial # = N73/285	Midden	**Wk-6648; 632±44**<br>Wk-6663; 730±43<br>Wk-6664; 715±38<br>Wk-6665; 735±39	**Charcoal: identified**<br>*Austrovenus stutchburyi*<br>*Paphies subtriangulata*<br>*Paphies australis*	609 cal BP<br>(564-657 BP)	-284±59<br>-298±56<br>-278±56	-287±41<br>$\chi^2_{2:0.05}=0.03<5.99$<br>gSD=10	McKinnon (p. 87)[9] suggested inbuilt age in the charcoal

Table S1 cont.

Site	Context	Details#	Material	Terrestrial statistics median age (68% prob. range)**	Δ*R* ¹⁴C years (68% prob.)	Δ*R* statistics	Reference/Comments
**Sites dating to between 600 and 650 cal BP (AD 1300 - 1350)**							
**Shag River Mouth Area C, Dune**<br>NZAA # = J43/2<br>Imperial # = S155/5	Layer 4	NZ-7761; 600±50<br>Wk-5433; 639±40<br>Wk-4930; 880±41<br>Wk-4970; 937±40<br>WK-4971; 865±39<br>Wk-5131; 1035±42<br>Wk-5434; 909±45<br>Wk-5435a; 988±38<br>Wk-5435b; 944±20<br>(OxA-12829; 617±25)<br>(OxA-12830; 572±24)<br>(OxA-13239; 523±23)<br>(NZ-7804; 747±38)<br>NZ-7805; 965±26<br>Wk-2362; 1010±50<br>Wk-2364; 1040±50<br>Wk-2410; 1020±50<br>Wk-2411; 990±45<br>Wk-2412; 980±45<br>Wk-2440; 1050±50<br>Wk-2441; 1070±45<br>Wk-2508; 1060±45<br>Wk-2632; 980±40<br>Wk-2751; 960±45<br>Wk-2752; 1040±45<br>Wk-2856; 980±40<br>Wk-2857; 1050±50<br>(OxA-12831; 1063±24)<br>(OxA-12832; 1090±25)<br>(OxA-12833; 1062±25)<br>(OxA-12834; 1035±24)<br>(OxA-12835; 992±24)<br>(OxA-13093; 1001±27)<br>(OxA-13240; 1058±23)	**Charcoal: identified**<br>**Moa bone (gelatin)**<br>*Thyrsites atun* (gelatin)<br>*Thyrsites atun* (gelatin)<br>*Thyrsites atun* (gelatin)<br>*Thyrsites atun* (gelatin)<br>*Thyrsites atun* (gelatin)<br>*Thyrsites atun* (gelatin)<br>*Thyrsites atun* (ultrafiltered gelatin)<br>Pigeon: Hemiphaga novaeseelandiae (ultrafiltered gelatin)<br>Pigeon: Hemiphaga novaeseelandiae (ultrafiltered gelatin)<br>Pigeon: Hemiphaga novaeseelandiae (ultrafiltered gelatin)<br>Haliotis iris<br>*Austrovenus stutchburyi*<br>*Austrovenus stutchburyi*<br>*Austrovenus stutchburyi*<br>*Austrovenus stutchburyi*<br>*Austrovenus stutchburyi*<br>*Austrovenus stutchburyi*<br>*Austrovenus stutchburyi*<br>*Austrovenus stutchburyi*<br>*Austrovenus stutchburyi*<br>*Paphies australis*<br>*Austrovenus stutchburyi*<br>*Mytilus edulis*<br>*Austrovenus stutchburyi*<br>*Austrovenus stutchburyi*<br>Shy mollymawk (Diomedea cauta)<br>Shy mollymawk (Diomedea cauta)<br>Shy mollymawk (Diomedea cauta)<br>Blue penguin (Eudyptula minor)<br>Blue penguin (Eudyptula minor)<br>Blue penguin (Eudyptula minor)<br>Shy mollymawk (Diomedea cauta)	600 cal BP<br>(544-630 BP)<br>$\chi^2_{1:0.05}=0.37<3.84$	-126 ± 52<br>-70 ± 52<br>-142 ± 50<br>28 ± 52<br>-98 ± 54<br>-19 ± 49<br>-62 ±38<br>(-260 ± 49)<br>-41 ± 41<br>3 ± 59<br>33 ± 59<br>13 ± 59<br>-16 ± 54<br>-26 ± 54<br>43 ± 59<br>64 ± 54<br>54 ± 54<br>-26 ± 50<br>-46 ± 54<br>34 ± 54<br>-26 ± 50<br>43 ± 59<br>-<br>-<br>-<br>-<br>-<br>-<br>-	-24±11<br>$\chi^2_{20:0.05}=24.46<31.41$<br>gSD=58	Anderson et al. (p. 60-69)[10]; Petchey (p. 149-152)[11]; Higham et al.[12].<br>Considered to be a short-lived midden deposit. NZ-7804 is an outlier (Anderson et al. 1996:66) and has been excluded from this evaluation.<br>OxA dates excluded from average Δ*R* calculation. See S1 text for details.
**Taputapuatea**<br>NZAA# = T11/914	Area 6 Layer P	**Wk-41163; 676±20**<br>**Wk-43228; 676±20**<br>Wk-41167; 1001±30	**Charcoal: identified**<br>**Charcoal: identified**<br>*Perna canaliculus*	602 cal BP<br>(562-646 BP)<br>$\chi^2_{1:0.05}=0.00<3.84$	-44 ± 42	-	Hoffmann (p. 30)[27]
**Watsons Beach, Area B**<br>NZAA # = H45/10<br>Imperial # = S172/55	Layer 2	**Wk-12966; 631±44**<br>Wk-12963; 988±37<br>Wk-12964; 1045±45<br>Wk-12965; 1018±39	**Moa eggshell**<br>“Mussel”<br>“Paua”<br>“Mussel”	598 cal BP<br>(543-634 BP)	-25 ± 55<br>32 ± 60<br>4 ± 56	2±33<br>$\chi^2_{2:0.05}=0.49<5.99$<br>gSD=29	Kirk (p. 82)[30]; Jacomb and Darmody (p. 54)[31]
**Shag River Mouth Area C, Dune**<br>NZAA # = J43/2<br>Imperial # = S155/5	Layer 7	**NZ-7755; 646±47**<br>NZ-7806; 1022±29	**Charcoal: identified**<br>*Haliotis iris*	602 cal BP<br>(550-639 BP)	-2 ± 52	-	Anderson et al. (p. 60-69)[10]
**Shag River Mouth Area C, Dune**<br>NZAA # = J43/2<br>Imperial # = S155/5	Layer 11	**NZ-7771; 660±46**<br>NZA-1175; 974±49	**Charcoal: identified**<br>*Haliotis iris*	603 cal BP<br>(555-640 BP)	-60 ± 64	-	Anderson et al. (p. 60-69)[10]
**Cabana Lodge**<br>NZAA # = T12/3<br>Imperial # = N49/3	Layer 3	**Wk-33850; 635±28**<br>Wk-33849; 1038±25<br>Wk-33698; 981±25	**Charcoal: identified**<br>*Pagrus auratus* (ultrafiltered gelatin)<br>*Pagrus auratus* (ultrafiltered gelatin)	605 cal BP<br>(550-630 BP)	24±38<br>-32±38	-4±28<br>$\chi^2_{1:0.05}=1.09<3.84$<br>gSD=40	Gumbley et al. (p. 45)[35]
**Wairau Bar**<br>NZAA # = P28/21	Area 4, Unit J, Oven Pit 1<br>Main occupation / Layer 4 midden	**NZA-34411; 667±25**<br>**NZA-34412; 641±25**<br>**NZA-34413; 638±25**<br>**NZA-34414; 618±25**<br>**NZA-34415; 642±25**<br>**NZA-34416; 646±30**<br>**NZA-34417; 649±25**<br>**NZA-34418; 669±25**<br>**NZA-34419; 654±25**<br>**NZA-34420; 676±25**<br>**NZA-34421; 655±25**<br>**NZA-38894; 637±25**<br>NZ-1837; 1029±41<br>Wk-4458; 1113±45<br>Wk-4459; 1091±41<br>Wk-4967; 1061±40	**Moa eggshell**<br>**Moa eggshell**<br>**Moa eggshell**<br>**Moa eggshell**<br>**Moa eggshell**<br>**Moa eggshell**<br>**Moa eggshell**<br>**Moa eggshell**<br>**Moa eggshell**<br>**Moa eggshell**<br>**Moa eggshell**<br>**Moa eggshell**<br>*Paphies australis*<br>*Austrovenus stutchburyi*<br>*Austrovenus stutchburyi*<br>*Austrovenus stutchburyi*	614 cal BP<br>(603-627 BP)<br>$\chi^2_{11:0.05}=4.57<19.68$	34 ± 46<br>118 ± 50<br>96 ± 46<br>66 ± 46	77±24<br>$\chi^2_{3:0.05}=1.77<7.81$<br>gSD=37	Jacomb et al.[15]; Higham et al.[14]
**Cross Creek**<br>NZAA # = T10/399<br>Imperial # = N40/260	Layer 9	**Wk-21642; 744±33**<br>Wk-21355; 1007±34<br>Wk-21363; 1092±34	**Nestor meridionallis (ultrafiltered gelatin)**<br>*Pagrus auratus* (ultrafiltered gelatin)<br>*Pagrus auratus* (ultrafiltered gelatin)	653 cal BP<br>(574-676 BP)	-97±45<br>-12±45	-54±32<br>$\chi^2_{1:0.05}=1.78<3.84$<br>gSD=60	Furey et al.[36]

Table S1 cont.

Site	Context	Details#	Material	Terrestrial statistics median age (68% prob. range)**	ΔR ^{14}C years (68% prob.)	ΔR statistics	Reference/Comments
**Sites older than 650 cal BP (older than AD 1300)**							
**Fyffes (Avoca Point)**<br>NZAA # = O31/30<br>Imperial # = S49/46	Occupation deposit, Sq 9-10	**NZ-2716; 840±60**<br>NZ-2718; 1183±29<br>NZ-2719; 1174±33	**Charcoal: identified**<br>*Turbo smaragda*<br>*Protothaca crassicosta*	722 cal BP (674-757)	2±58<br>-7±60	-2±42<br>$\chi^2_{1:0.05}$=0.01<3.84<br>gSD=6	Trotter[37]

^Information on each site location can be found at http://www.archsite.org.nz

** All dates have been calibrated using OxCal v.4.3.2.

$^+$ Chi square test and gSD (Gaussian standard deviation).

* Dates initially reported as "modern" following the recommendations of Stuiver and Polach[38] for reporting radiocarbon ages less than 200 BP.

# All Waikato University dates (Wk-) have been checked against laboratory files. In some cases, radiocarbon ages were originally reported rounded to the nearest 10 years following the recommendations of Stuiver and Polach[38]. Where possible, this has been removed and dates may therefore differ from published reports.

Brackets = individual dates removed from ΔR average (see S1 text for discussion).

Table S2. Change in ΔR over time as determined by black coral (Tasmania) and New Zealand archaeological pairs for use with Marine13[39]

Cal BP (AD)	Black Coral Dataset			NZ Archaeological Dataset		
	No.	Mean and error (^{14}C years)	Chi-square statistics	No.	Mean and error (^{14}C years)	Chi-square statistics
0-50* (AD 1900-1500)	8	29±10	$\chi^2_{7:0.05}$ =17.88<14.07; gSD=47	2	71 ± 31	$\chi^{21}_{1:0.05}$=3.06<3.84; gSD=79
50-100 (AD 1850-1900)	3	45±15	$\chi^2_{2:0.05}$=7.3<5.99; gSD=12			
100-150 (AD 1800-1850)	8	64 ± 14	$\chi^2_{7:0.05}$=1.22<14.07; gSD=19			
150-200 (AD 1750-1700)	7	22 ± 15	$\chi^2_{6:0.05}$=9.88<12.59; gSD=60			
200-250 (AD 1700-1750)	4	99 ± 30	$\chi^2_{3:0.05}$=2.94<7.81; gSD=59			
250-300 (AD 1650-1700)	7	29 ±18	$\chi^2_{6:0.05}$=4.23<12.59; gSD=45			
300-350 (AD 1600-1650)	4	21±21	$\chi^2_{3:0.05}$=20.60<7.81; gSD=104	1	-26±73	
350-400 (AD 1550-1600)	3	-11±24	$\chi^2_{2:0.05}$=1.34<5.99; gSD=35	8	-19±15	$\chi^2_{7:0.05}$=6.95<14.07; gSD=40†
400-450 (AD 1500-1550)	3	-4±20	$\chi^2_{2:0.05}$=19.96<5.99; gSD=97	11	-34±13	$\chi^2_{10:0.05}$=19.46<18.31; gSD=65
450-500 (AD 1450-1500)	9	-56±18	$\chi^2_{8:0.05}$=7.6<15.51; gSD=53			
500-550 (AD 1400-1450)	4	-61±28	$\chi^2_{3:0.05}$=10.9<7.81; gSD=96	4	40±18	$\chi^2_{3:0.05}$=3.42<7.81; gSD=39
550-600 (AD 1350-1300)	1	-198±50	-	6	-172±17	$\chi^2_{5:0.05}$=28.91<11.07; gSD=92†
600-650 (AD 1300-1350)	-	-	-	7	-15±17	$\chi^2_{6:0.05}$=1.43<12.59; gSD=24†
650-700 (AD 1250-1300)	3	-42±25	$\chi^2_{2:0.05}$=0.01<5.99; gSD=44	1	-2±42	
700-750 (AD 1200-1250)	1	-3±36	-			

*Grey boxes are 50-year blocks where there is rapid change and significant ΔR instability.

† Average value excludes ΔR from Wairau Bar, Ponui Island Site 333 (Layer B), Ponui Island Site 95 (Layer D) and Pleasant River Area D (Layer 4) (see S1 text for discussion).

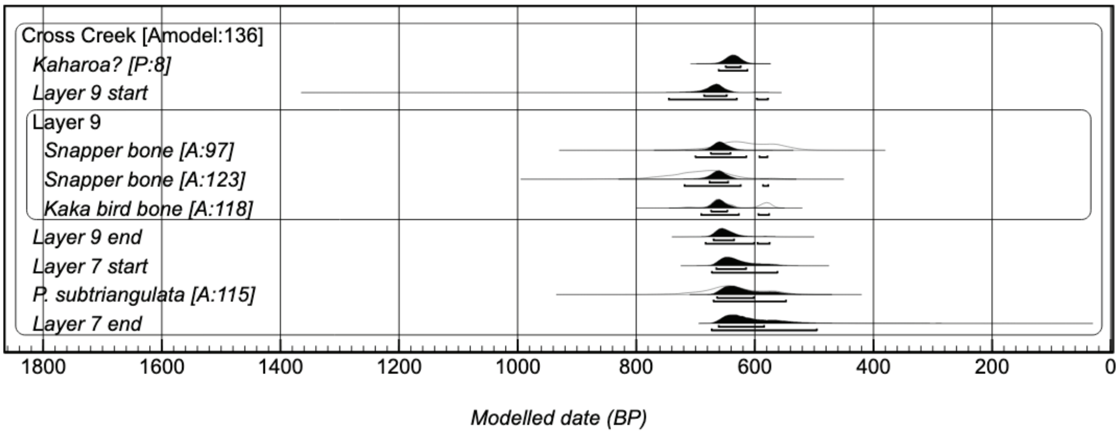

a.

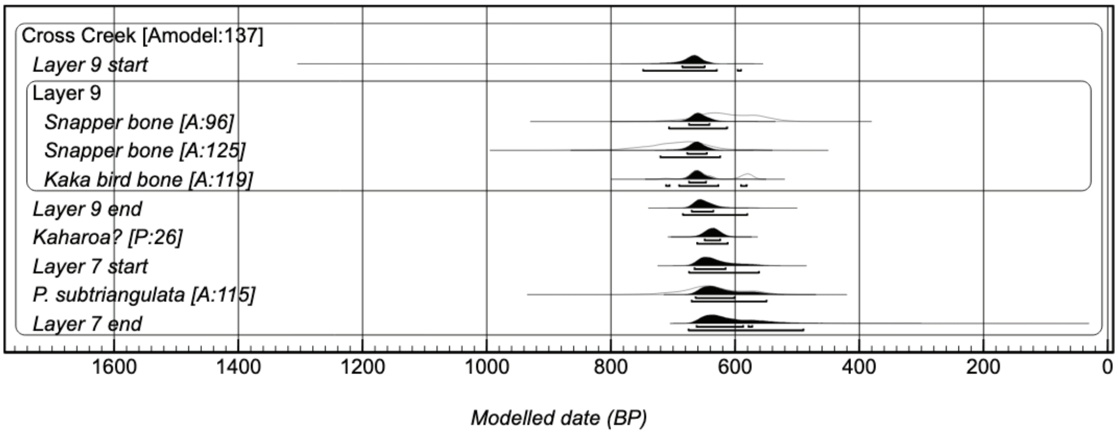

b.

Fig. S2: Calibrated results for Cross Creek, layers 7 to 9 using the black coral ΔR of -42 ± 44 ^{14}C yrs (see Table S2) for the period between 600 and 700 cal BP (OxCal code given in S4 text). The position of the Kaharoa Tephra has been "questioned". P= probability that this determination occupies that position. Note: in this example the lowest probability (P=8) is obtained if the tephra deposition is placed prior to Layer 9 (a) as opposed to between layers 7 and 9 (P=26) (b).

4. OxCal code for Cross Creek Fig. S2

```
Plot()
 {
  Curve("Marine13","Marine13.14c");
  Sequence("Start Layer 9")
  {
   Boundary("Layer 9 start");
   Phase("Layer 9")
   {
    Delta_R("LocalMarine",-42,44);
    R_Date("Snapper", 1007, 34);
    R_Date("Snapper2", 1092, 34);
    Curve("SHCal13","SHCal13.14c");
    R_Date("Kaka bird bone", 744, 33);
   };
   Boundary("Layer 9 end");
   C_Date("Kaharoa Tephra", 1314, 12)
   {
    Outlier("?");
   };
   Boundary("Layer 7 start");
   Curve("Marine13","Marine13.14c");
   Delta_R("LocalMarine",-42,44);
   R_Date("P. subtriangulata", 1035, 28);
   Boundary("Layer 7 end");
  };
 };
```

5. References

1. Schmidt, M. Radiocarbon dating New Zealand prehistory using marine shell. *BAR International Series, 842* (John and Erica Hedges and Archaeopress) (2000).
2. Bickler, S., *et al.* Omaha Beach: Final Archaeological Report. (Clough & Associates, Auckland, 2003). Avaliable http://www.clough.co.nz/monographs/clough_omaha_beach.pdf (2003).
3. Allingham, B.J. Preliminary report on salvage excavations at Tumbledown Bay, Banks Peninsula. NZHPT permit 1987/9. Available https://dl.heritage.org.nz/greenstone3/library/collection/pdf-reports/document/Allingham8 (1988).
4. Challis, A.J. Ka pakihi whakatekateka o waitaha: The archaeology of Canterbury in Maori times. Science & Research series 89 (Department of Conservation, Wellington). Available https://www.doc.govt.nz/Documents/science-and-technical/sr89.pdf (1995).
5. Anderson, A.J. The chronology of colonization in New Zealand. *Antiquity* **65**, 767-95 (1991).
6. Higham, T.F.G. Radiocarbon dating the prehistory of New Zealand. (PhD Thesis, University of Waikato, 1993).
7. Smith, I.W.G. Settlement permanence and function at Pleasant River Mouth, east Otago, New Zealand. *New Zealand Journal of Archaeology* **19**, 27-79 (1997).
8. Walter, R., Jacomb, C., & Brooks, E. Final report on archaeological excavations at Cooks Cove Z17/311, Tolaga Bay, East Coast, North Island. (Southern Pacific Archaeological Research 22. Available https://www.otago.ac.nz/spar/publications/otago634090.pdf (2010).
9. McKinnon, H. Investigation of a hard water effect at Aotea Harbour, North Island, New Zealand. (M.Sc. Thesis, University of Waikato, 1999).
10. Anderson, A., Smith, I., & Higham, T. Radiocarbon chronology in *Shag River Mouth: The archaeology of an early southern Maori village* (eds. Anderson, A., Allingham, B., & Smith, I.) 276-91 (Australian National University Press, 1996).
11. Petchey, F. Radiocarbon Analysis of a novel bone sample type: Snapper and barracouta bone from New Zealand archaeological sites. (D.Phil Thesis, University of Waikato, 1998).
12. Higham, T.F.G., Anderson, A., Bronk Ramsey, C., & Tompkins C. Diet-derived variations in radiocarbon and stable isotopes: A case study from Shag River Mouth, New Zealand. *Radiocarbon* **47**, 367–375 (2005).
13. Walter, R., Buckley, H., Jacomb, C., & Matisoo-Smith, E. Mass migration and the Polynesian settlement of New Zealand. *J World Prehist*, **30**:351-376;10.1007/s10963-017-9110-y (2017).
14. Jacomb, C., *et al.* High-precision dating and ancient DNA profiling of moa (Aves: Dinornithiformes) eggshell documents a complex feature at Wairau Bar and refines the chronology of New Zealand settlement by Polynesians. *Journal of Archaeological Science* **50**, 24-30 (2014).
15. Higham, T., Anderson, A., & Jacomb, C. Dating the first New Zealanders: the chronology of Wairau Bar. *Antiquity* **73**:420-27 (1999).
16. Holdaway, R.N. et al. An extremely low-density human population exterminated New Zealand moa. *Nature Communications* **5**(5436);10.1038/ncomms6436 |(2014).
17. McFadgen, B.G. Beach ridges, breakers and bones: Late Holocene geology and archaeology of the Fyffe site, S49/46, Kaikoura Peninsula, New Zealand. *Journal of the Royal Society of New Zealand* **17**, 381-394;10.1080/03036758.1987.10426479 (1987).

18. Anderson, A.J. *Prodigious Birds. Moas and Moa-Hunting in Prehistoric New Zealand.* (Cambridge University Press, 1989).
19. McFadgen, B.G., Knox, F.B., & Cole, T.R.L. Radiocarbon calibration curve variations and their implications for the interpretation of New Zealand prehistory. *Radiocarbon* **36**, 221-236 (1994).
20. Culleton, B.J., Kennett, D.J., Ingram, B.L., Erlandson, J.M., & Southon J.R. Intrashell radiocarbon variability in marine mollusks, *Radiocarbon* **48**, 387–400 (2006).
21. Holmquist, J.R., et al. Marine radiocarbon reservoir values in southern California estuaries: Interspecies, latitudinal, and interannual variability. *Radiocarbon* **57**, 449–458;10.2458/azu_rc.57.18389 (2015).
22. Chiswell, S.M., & Schiel, D.R. Influence of a long-shore advection and upwelling on coastal temperature at Kaikoura Peninsula, New Zealand. *New Zealand Journal of Marine and Freshwater Research* **35**, 307-317;10.1080/00288330.2001.9517000 (2001).
23. Doelman, T. Te awa a korako: The archaeology and ethnohistory of Te Kiri Kiri. (MA Thesis, University of Auckland, 1995).
24. Bedford, A.H. & Allen, H. When is a shell midden not just a shell midden? Excavations on the Puriri River, Hauraki Plains. *Archaeology in New Zealand* **36**,120-134 (1993).
25. Furey, L., Emmitt, J., & Wallace R. Matakawau Stingray Point Pa excavation, Ahuahu Great Mercury Island 1955-56. *Records of the Auckland Museum* **52**, 39-57 (2017).
26. Fredericksen, C.F.K., & Visser E. Excavation of the Cryers Road site (R11/1519), East Tamaki, Auckland. *Archaeology in New Zealand* **31**, 233-250 (1988).
27. Hoffmann, A. Investigation of archaeological site T 11/914, Taputapuatea Stream, Whitianga, Mercury Bay – Stage 4E. (Unpublished Report for: Pacific Estates Limited & Heritage New Zealand, 2014).
28. Barber, I. Sea, Land and fish: Spatial relationships and the archaeology of South Island Maori fishing. *World Archaeology* **35**, 434-448 (2003).
29. Campbell, M., et al. The Torpedo Bay excavations: Vol 1, the pre-European Maori site (HPA authority 2009/275). (Report to Heritage New Zealand Pouhere Taonga and The New Zealand Defence Force. Available http://www.cfgheritage.com/17_0773torpedobay1.pdf (2018).
30. Kirk, F. Beyond reasonable doubt? Testing the cultural origin of bird bone recovered from archaeological contexts. (MA Thesis, University of Otago, 2010).
31. Jacomb, C., & Darmody, R. Interim report on excavations at Watson's Beach (H45/10); an early coastal Otago archaeological site. *Archaeology in New Zealand* **45**, 47-58 (2002).
32. Petchey, F. Radiocarbon dating fish bone from the Houhora archaeological site, New Zealand. *Archaeology in Oceania* **35**, 104-15 (2000).
33. Furey, L. Houhora: A fourteenth century Maori village in Northland. *Bulletin of the Auckland Museum*, **19** (2002).
34. Walter, R., Jacomb, C., & Bowron-Muth, S. Colonisation, mobility and exchange in New Zealand prehistory. *Antiquity* **84**, 497–513 (2010).
35. Gumbley, W., Turner, M., & James Lee T. The Cabana site (T12/3), Whangamata: Results of the 2007 investigation. Available https://dl.heritage.org.nz/greenstone3/library/collection/pdfreports/document/Gumbley90 (2014).
36. Furey, L., Petchey, F., Sewell, B., & Green, R. New observations on the stratigraphy and radiocarbon dates at the Cross Creek site, Opito, Coromandel Peninsula. *Archaeology in New Zealand* **51**, 46-64 (2008).

37. Trotter, M.M., Archaeological investigations at Avoca Point, Kaikoura. *Records of the Canterbury Museum* **9**, 277-288 (1980).
38. Stuiver, M., & Polach, H.A., Discussion: Reporting ^{14}C data. *Radiocarbon* **19**, 355–63 (1977).
39. Reimer, P., *et al.* IntCal13 and Marine13 radiocarbon age calibration curves, 0–50 000 years cal BP. *Radiocarbon* **55**, 1869–1887 (2013).
